# Epilithic Microbial Community Functionality in Deep Oligotrophic Continental Bedrock

**DOI:** 10.3389/fmicb.2022.826048

**Published:** 2022-03-01

**Authors:** Maija Nuppunen-Puputti, Riikka Kietäväinen, Mari Raulio, Aino Soro, Lotta Purkamo, Ilmo Kukkonen, Malin Bomberg

**Affiliations:** ^1^VTT Technical Research Centre of Finland Ltd., Espoo, Finland; ^2^Geological Survey of Finland, Espoo, Finland; ^3^European Chemicals Agency (ECHA), Helsinki, Finland; ^4^Department of Physics, University of Helsinki, Helsinki, Finland

**Keywords:** crystalline bedrock, deep biosphere, Fennoscandian Shield, sessile microbial communities, the Outokumpu deep drill hole, metagenome-assembled genomes, microbe-mineral interactions, sulfate reduction

## Abstract

The deep terrestrial biosphere hosts vast sessile rock surface communities and biofilms, but thus far, mostly planktic communities have been studied. We enriched deep subsurface microbial communities on mica schist in microcosms containing bedrock groundwater from the depth of 500 m from Outokumpu, Finland. The biofilms were visualized using scanning electron microscopy, revealing numerous different microbial cell morphologies and attachment strategies on the mica schist surface, e.g., bacteria with outer membrane vesicle-like structures, hair-like extracellular extensions, and long tubular cell structures expanding over hundreds of micrometers over mica schist surfaces. Bacterial communities were analyzed with amplicon sequencing showing that *Pseudomonas*, *Desulfosporosinus, Hydrogenophaga*, and *Brevundimonas* genera dominated communities after 8–40 months of incubation. A total of 21 metagenome assembled genomes from sessile rock surface metagenomes identified genes involved in biofilm formation, as well as a wide variety of metabolic traits indicating a high degree of environmental adaptivity to oligotrophic environment and potential for shifting between multiple energy or carbon sources. In addition, we detected ubiquitous organic carbon oxidation and capacity for arsenate and selenate reduction within our rocky MAGs. Our results agree with the previously suggested interaction between the deep subsurface microbial communities and the rock surfaces, and that this interaction could be crucial for sustaining life in the harsh anoxic and oligotrophic deep subsurface of crystalline bedrock environment.

## Introduction

Deep life in continental bedrock inhabits both groundwater and all water-covered rock surfaces and fractures. Microbes attach to rock surfaces, remaining as single sessile cells or forming microcolonies and biofilms (Wanger et al., 2006; Escudero et al., 2018). Deep continental microbial biomass has been estimated to constitute up to 19% of the Earth’s biomass (McMahon and Parnell, 2014; Bar-On et al., 2018; Magnabosco et al., 2018). Flemming and Wuertz (2019) estimated that 20–80% of the deep subsurface microbial biomass resides in biofilms (Flemming and Wuertz, 2019), which means that the previous biomass evaluations may be underestimated. However, challenges related to difficulty of retrieving native deep subsurface biofilm samples and how biofilm are defined for these calculations remain (Bar-On and Milo, 2019; Flemming and Wuertz, 2019). In general, dense biofilms in deep anaerobic biosphere may not form and all mineral attached single cells are considered as biofilm in these estimations (Moser et al., 2003; Wanger et al., 2006; Magnabosco et al., 2018; Flemming and Wuertz, 2019). Attached cell densities on various deep mineral surfaces have been shown to exceed planktic cell counts by up to five orders of magnitude in the Deep Mine Microbial Observatory (DeMMO), United States (Casar et al., 2020), and by at least two orders of magnitude in the South African deep mines (Wanger et al., 2006). In comparison, microbial cell abundance in crushed deep rock core from the Deccan traps, India, have been shown to reach nearly 10^5^ cells per gram estimated by qPCR (Dutta et al., 2018).

Microbial life in deep continental subsurface expands to several kilometers depth (e.g., Pedersen, 1997; Moser et al., 2005; Gihring et al., 2006; Trimarco et al., 2006; Purkamo et al., 2020; Dai et al., 2021). Recently, rock hosted microorganisms in drilled rock cuttings have been detected down to 4.4 km depth in Otaniemi, Finland (Purkamo et al., 2020), and within drilled rock cores down to 4.8 km depth in eastern China (Dai et al., 2021). Multi-species biofilms covering deep subsurface rock matrix have been shown to also contain polysaccharides, proteins, and lipids (Escudero et al., 2018). Furthermore, energy rich deep subsurface minerals have been shown to accumulate more biomass and attached microbial communities than energy poor minerals or inert glass surfaces (Casar et al., 2020). In addition, the attached rock surface microbial communities can remarkably differ from the planktic communities in surrounding groundwater (Momper et al., 2017). Restricting research focus solely on planktic lifestyle in the deep subsurface may hinder our understanding of life sustaining microbial processes linked to vital interaction with minerals.

A biofilm lifestyle enables chemolithotrophic microbes to utilize mineral surfaces for their metabolic actions (Gorbushina, 2007; Gadd, 2010; Ng et al., 2016; Casar et al., 2021b). Microbes are able to weather rock by secreting organic acids and can use mineral surfaces as a source of nutrients and trace elements (Cockell and Herrera, 2008; Uroz et al., 2009; Dong, 2010; Finlay et al., 2020; Samuels et al., 2020). In addition, in an oligotrophic environment that lacks readily available organic carbon sources, mineral surfaces may offer microbial cells a potential electron source or sink, widening their alternatives for life sustaining energy generation pathways (Dong, 2010; Ng et al., 2016; Shi et al., 2016). In deep continental bedrock, iron- and manganese-bearing minerals may enable external electron transfer (EET) for microorganisms using sulfur oxidation to obtain energy (Casar et al., 2020). Deep rock core bacterial isolates have revealed that the microbiome hosts wide genetic potential ranging from sulfate reduction, methanogenesis, and fermentation to acetogenesis in the Iberian Pyrite Belt (IPB) (Leandro et al., 2018). Further IPB rock core incubations indicated potential for active microbial gas production (H_2_, CH_4_, and CO_2_) in the deep subsurface (Sanz et al., 2021).

Mica schist naturally present in the Outokumpu deep subsurface contain carbon-, iron-, sulfur-, and phosphorus-bearing minerals, and could be a potential source for carbon and nutrients for the microbes (Västi, 2011). *In situ*-grown biofilms also revealed diverse fungal and bacterial communities co-inhabiting mica schist surfaces after a 6-month incubation at 500 and 967 m depth in the Outokumpu Deep Drill Hole, Finland (Nuppunen-Puputti et al., 2021). Mineral surfaces enhance interaction between microbial cells, which may cross kingdom boundaries (Drake et al., 2017; Nuppunen-Puputti et al., 2021). Survival in the oligotrophic deep subsurface depends on efficient nutrient recycling and senescent biofilms may help to sustain deep fungal communities that feed on carbonaceous remains of rock surface biofilms (Drake et al., 2017). In addition, fossil records show that fungi may funnel H_2_ to sulfate-reducing bacteria in deep subsurface biofilms (Drake et al., 2017).

Microbial communities in the Outokumpu deep groundwater have been shown to grow in the presence of acetate (Purkamo et al., 2017) and to incorporate carbon from acetate and carbonate into biomass (Bomberg et al., 2017; Nuppunen-Puputti et al., 2018). In addition, metagenomic and functional gene detection has indicated that despite the oligotrophic and anoxic conditions in the Outokumpu deep subsurface, heterotrophic lifestyles prevail, and biological sulfate reduction is an important process in the sulfate rich parts of Outokumpu deep subsurface (Purkamo et al., 2013, 2014; Nyyssönen et al., 2014). Methanogenic archaea have been found, but less frequently (Purkamo et al., 2013, 2016; Nyyssönen et al., 2014).

Our aim was to study the development of microbial communities from Outokumpu on rock surfaces over several years and to test research methods for sessile microbial communities. Here, we used mica schist as a surface, and potential carbon and nutrient source to support microbial biofilm formation in anaerobic deep groundwater microcosm enrichments. We visualized microbial cell attachment strategies on mica schist surfaces through time series and aimed to estimate the impact of microbial diversity on core nutrient cycling. We characterized the microbial communities through amplicon sequencing and analyzed rock surface microbial community metagenomes developed over 8 and 40 months. Our results show that sessile microbial communities developed in relatively short microcosm experiments. We also show that microcosm and *in situ* enrichment are different but complement each other in the oligotrophic crystalline bedrock.

## Materials and Methods

### Site Description and Sampling

The study site is located at the Fennoscandian Shield in the Outokumpu area, Finland (62.72 N, 29.07E). The drilling of scientific Outokumpu deep drill hole was finalized in 2005 and it belongs to the International Continental Drilling Program infrastructure (ICDP)^1^ (Kukkonen, 2011). The bedrock lithology, sampling techniques and campaigns, hydrogeology, water chemistry, gas composition, and microbial communities have been widely described in previous studies (Ahonen et al., 2011; Itävaara et al., 2011; Kukkonen et al., 2011; Kietäväinen et al., 2013, 2014, 2017; Purkamo et al., 2013, 2014, 2016, 2017; Nyyssönen et al., 2014; Rajala et al., 2015a; Sharma et al., 2016; Bomberg et al., 2017; Kietäväinen, 2017; Nuppunen-Puputti et al., 2018, 2021). The main rock type at our sampling depth is mica schist, which was also used in our microcosms (Lahtinen et al., 2010; Västi, 2011; Supplementary Table 1). Groundwater at the sampling depth belongs to alkaline Na-Ca-Cl rich water type II expanding from 300 to 1300 m below surface level (Kietäväinen et al., 2013). Detailed geochemical data of the groundwater used in the microcosms is presented in Supplementary Table 1. The highest determined gas-to-water ratio (up to 1.4 L gas per 1 L of water) was detected at the 500 m depth in Outokumpu groundwater with gas composition constituting mainly of methane and nitrogen, with minor proportion of ethane (C_2_H_6_), helium, argon, propane (C_3_H_8_), and hydrogen (Kietäväinen et al., 2013; Kietäväinen, 2017). Cell count in the fracture fluid at 500 m depth has been estimated to be 5.7 × 10^4^ ml^–1^ (Purkamo et al., 2013).

Sampling of fluid from the fracture zone at 500 m depth was performed in October 2010, as previously described in detail by Rajala et al. (2015a). The fracture zone was isolated from the rest of the borehole by inflatable packers spaced 24 m apart, above and below the fracture zone. The packer isolated drill hole compartment was purged for 21 days, i.e., five times the volume of the isolated drill hole section, before sample water was collected. Fracture fluid was pumped *via* a sterile polyamide tube from the isolated fracture zone at 500 m depth directly into an anaerobic field chamber (GB-2202-S, MBRAUN, Garching, Germany) under constant N_2_-flow. Anoxic conditions were further ensured by applying 2–3 Anaerocult A anaerobic gas generator packs (Merck, Darmstadt, Germany) in the chamber at all times and changing them daily. Anaerotest indicator strips (Merck, Darmstadt, Germany) were also used to monitor the anaerobic chamber for any oxygen contamination. Fracture fluid was first collected into acid washed and autoclaved 2000-ml Schott-bottles (Duran Group, Wertheim/Main, Germany) and then further divided into 80 ml microcosms. Microcosms were kept at + 4°C until the start of the incubation at the laboratory at + 11°C corresponding to the *in situ* temperature at 500 m depth (Ahonen et al., 2011).

### Mica Schist Microcosms

Triplicate microcosms containing anaerobic fracture fluid, crushed mica schist (MSC), or mica schist slides (MSS) were prepared for subsequent DNA and RNA extraction and SEM. Crushed mica schist was prepared as described in Nuppunen-Puputti et al. (2021) at the Geological Survey of Finland (GTK). Mica schist from the drill core corresponding to the sampling depth was crushed with a ball mill and sieved in order to collect small < 5-mm-sized fraction for incubations. Mica schist slides (size 32 × 16 × 4 mm) were cut with a rock saw, polished, and ultrasonicated in distilled water as a final wash step. Mica schist crush was pre-sterilized overnight at 160°C in a heat sterilizer cabinet in a glass jar closed with aluminum foil. Three grams of mica schist crush was weighted into headspace-bottles (i.e., microcosms), capped with blue butyl rubber caps, crimped with aluminum crimp caps, and sterilized for a second time by autoclaving at 121°C for 15 min. Microcosms containing mica schist slides were prepared in acid-washed and heat-sterilized borosilicate bottles (Schott, Duran Group, Wertheim/Main, Germany) that were closed with black butyl rubber stoppers (20 mm thickness) and open top screw caps (Schott). Fracture fluid samples (80 ml) were subsequently added to the microcosm bottles. Control microcosms containing sterile distilled water and crushed glass, mica schist slides, or crushed mica schist were prepared at the field site and incubated with the microcosm enrichments. The incubation time for the first set of microcosms was 8 months and that for the second batch was 40 months (Table 1). At the end of the incubation, water phase from the mica schist bottles was filtered onto Sterivex-filters (Millipore, Billerica, MA, United States). Sessile microbial communities were detached from the surfaces as described in Nuppunen-Puputti et al. (2021). In detail, 5 ml of PBS-buffer with 5 μl of Tween 20 (Bio-Rad, Hercules, CA, United States) was added to the crushed mica schist and 10 ml of PBS containing 10 μl of Tween 20 to mica schist slides. Then, samples were shaken at 150 rpm for 20 min and further ultrasonicated for 3 min. The detached biomass was collected onto second Sterivex-filter units. These second filters were further processed in the same extraction tube as the remaining crushed mica schist or glass. For negative control samples, distilled water and glass or mica schist were extracted as one collective sample.

**TABLE 1:** List of samples and their Sample IDs, type of microcosm, incubation time point, and sample type.

Time 1 (8 months)			Time 2 (40 months)			Time 1, Time 2
Sample ID	Microcosm	Type	Sample ID	Microcosm	Type	Analysis
MSS1neg	Mica schist slide	Control	MSS2neg	Mica schist slide	Control	iSeq, SEM
MSS1A	Mica schist slide	Mica schist	MSS2A	Mica schist slide	Mica schist	iSeq, SEM, metagenome
MSS1B	Mica schist slide	Mica schist	MSS2B	Mica schist slide	Mica schist	iSeq, SEM, metagenome
MSS1C	Mica schist slide	Mica schist	MSS2C	Mica schist slide	Mica schist	iSeq, SEM, metagenome
MSS1AW	Mica schist slide	Water	MSS2AW	Mica schist slide	Water	iSeq, SEM
MSS1BW	Mica schist slide	Water	MSS2BW	Mica schist slide	Water	iSeq, SEM
MSS1CW	Mica schist slide	Water	MSS2CW	Mica schist slide	Water	iSeq, SEM
MSC1neg	Mica schist crush	Control	MSC2neg	Mica schist crush	Control	iSeq, SEM
MSC1A	Mica schist crush	Mica schist	MSC2A	Mica schist crush	Mica schist	iSeq, SEM, metagenome
MSC1B	Mica schist crush	Mica schist	MSC2B	Mica schist crush	Mica schist	iSeq, SEM, metagenome
MSC1C	Mica schist crush	Mica schist	MSC2C	Mica schist crush	Mica schist	iSeq, SEM, metagenome
MSC1AW	Mica schist crush	Water	MSC2AW	Mica schist crush	Water	iSeq, SEM
MSC1BW	Mica schist crush	Water	MSC2BW	Mica schist crush	Water	iSeq, SEM
MSC1CW	Mica schist crush	Water	MSC2CW	Mica schist crush	Water	iSeq, SEM
G1neg	Glass	Control	G2neg	Glass	Control	iSeq
G1A	Glass	Glass	G2A	Glass	Glass	iSeq
G1B	Glass	Glass	G2B	Glass	Glass	iSeq
G1C	Glass	Glass	G2C	Glass	Glass	iSeq
G1AW	Glass	Water	G2AW	Glass	Water	iSeq
G1BW	Glass	Water	G2BW	Glass	Water	iSeq
G1CW	Glass	Water	G2CW	Glass	Water	iSeq

*Incubation time was 8 months for Time 1 and 40 months for Time 2. Abbreviations in Sample ID are as follows: G = Glass, MSS = Mica schist slide, MSC = Mica schist crush, W = Water phase from the microcosm collected on filter prior the wash step, 1, 2 = Time point, A, B, C = replicate incubations, neg = collective negative control sample with distilled water and sterile mica schist/glass. In addition, throughout the manuscript, the coding of Time 1 samples with c in front is used to discriminate cDNA samples from DNA samples listed in this table.*

### DNA Extraction, RNA Extraction, and cDNA

DNA was extracted with the PowerWater DNA isolation kit (MoBio Laboratories, Inc., Carlsbad, CA, United States) following the extraction protocol of the manufacturer. Extraction blank control was included in order to estimate contamination sources from the kit chemicals or extraction procedure. DNA was eluted into 50 μl of the PW6 elution buffer included in the kit. DNA was stored at –80°C until further analysis. The concentration of extracted DNA and its quality was estimated with a NanoDrop-1000 spectrophotometer (NanoDrop Technologies Inc., Wilmington, DE, United States). RNA was isolated from the Time 1 samples using the PowerWater RNA isolation kit (MoBio Laboratories, Inc., Carlsbad, CA, United States) according to the manufacturer’s protocol. RNA was eluted into 50 μl of the PW8 elution buffer included in the kit. RNA was stored at –80°C. RNA was treated with DNase as duplicate 27.5-μl reactions containing 20 μl of sample, 2.5 μl of the reaction buffer, and 2.5 μl of the DNase RQ1 (Promega, Madison, WI, United States). First mixture was incubated at 37°C for 30 min, followed by addition of 2.5 μl DNase stop solution and end incubation at 65°C for 10 min. The reverse transcription for RNA was performed with RT-PCR. A reaction mixture containing 0.5 μl of random primers, 1 μl of 10 mM dNTP (Thermo Fisher Scientific, Vantaa, Finland), and 11.5 μl of the DNase-treated RNA sample was incubated for 5 min at 65°C, followed by cooling on ice. The following reagents were added to the mixture: 4 μl of 5 × reaction buffer, 1 μl of DTT, 1 μl of RNase inhibitor and 1 μl of the SuperScript III reverse transcriptase (Invitrogen, Carlsbad, CA, United States). RT-PCR was carried out on an Eppendorf Mastercycler incubation at 25°C for 5 min, incubation at 50°C for 60 min, and final inactivation at 70°C for 15 min. The cDNA was kept at –80°C until further analysis.

### Amplicon Library Preparation for iSeq100

First, bacteria, archaea, and fungi targeting PCRs were performed with primers containing Illumina iSeq adapters. Bacteria were amplified with v3v4 targeting primers Bact_341F/Bact_805R (Herlemann et al., 2011), fungi with ITS1 targeting primers ITS1 and ITS2 (White et al., 1990; Gardes and Bruns, 1993), and archaea with v4 targeting primers S-D-Arch-0349-a-S-17/S-D-Arch-0787-a-A-20 (Klindworth et al., 2013). Duplicate 25-μl reactions for each sample was used. PCR mastermix included 12.5 μl of MyTaq Red Mix (Bioline, London, United Kingdom), 1 μl of each primer (20 μM), 8.5 μl of molecular grade water, and 2 μl of the template. Amplification was performed with PCR program that begun with 3 min at 95°C, then 40 cycles at 95°C, at 57°C and at 72°C (15 s each), followed by final elongation for 30 s at 72°C, and cooling to 4°C. PCR products were checked with agarose gel electrophoresis. Duplicate PCR products were pooled together to a total volume of 40 μl, followed by the purification step with NucleoMag NGS Clean-up and Size Select beads (32 μl per reaction) (Macherey-Nagel, Düren, Germany). Purification was performed in 4ti-0110 96-well plate (4titude, Surrey, United Kingdom) and NucleoMag SEP magnetic plate (Macherey-Nagel, Düren, Germany). Steps included double wash with 200 μl of 80% ethanol, desiccation for 10 min, and final elution with 52.5 μl of 10 mM Tris buffer, pH 8.5 (bioPLUS Buffers and Reagents, Dublin, Ohio, United States).

Samples were indexed using the Nextera XT version 2 kit (Illumina, Inc., San Diego, CA, United States) Set C and D for bacteria and fungi, respectively, in 25 μl reaction volumes containing 1 × MyTaq Red HS mastermix (Bioline, London, United Kingdom), 2.5 μl of each index, 1 μl of template, and 5 μl of molecular grade water (Sigma-Aldrich, St Louis, MO, United States). Index-PCR was performed with Eppendorf Mastercycler (Eppendorf, Hamburg, Germany). Index-PCR was initiated with 95°C for 30 s, followed by 8 cycles at 95°C for 30 s, at 57°C for 30 s, and at 72°C for 30 s, followed by final elongation at 72°C for 5 min and cooling to 4°C. The index-PCR products were purified with a similar protocol as described above with the exception that magnetic beads were used in 1:1 volume and final elution to 13 μl with 10 mM Tris, pH 8.5. The attachment of indexes and concentration of libraries were determined with qPCR performed using the JetSeq Hi-ROX Library Quantification Kit (Bioline, London, United Kingdom) and 1:100,000 sample dilution. The reaction volume was 10 μl. Kit standard series was used as a reference for the calculation, and a correction factor according to the expected lengths of the amplicons was applied. The qPCR program initiated with denaturation at 95°C for 2 min, followed by repeated 35 cycles of 95°C for 5 s and 60°C for 60 s, and finally followed by melting curve analysis and cooling to 40°C. Libraries were then individually diluted to equal concentration and combined into either bacterial or fungal library mixes separately. The combined library mixes were run through gel electrophoresis in 1 × SB buffer and 1% SB-gel, 120 V for 1 h, the correct size products were excised from the gel carefully avoiding any shorter fragments, and purified with the XS gel purification kit (Macherey-Nagel, Düren, Germany) according to the manufacturer’s protocol. The final purified libraries were eluted twice to a final volume of 12 μl. The concentration of the final purified libraries was determined as described above, pooled equimolarly to 115 pM concentration including 5% of PhiX Control V3 (Illumina, Inc., San Diego, CA, United States) to add diversity to samples, and 20 μl of the library sample was loaded into an iSeq v2 cassette (Illumina, Inc., San Diego, CA, United States).

### Metagenomic Sequencing

DNA samples for metagenomic sequencing were first whole genome amplified using the Illustra GenomiPhi™ V3 DNA Amplification Kit (GE Healthcare, Buckinghamshire, United Kingdom) according to the manufacturer’s protocol. A 2-μl sample template and 8 μl of molecular grade water (Sigma-Aldrich, St Louis, MO, United States) were mixed and then further amended with 10 μl of denaturation buffer. Samples were denaturated for 3 min at 95°C on Eppendorf MasterCycler (Eppendorf, Hamburg, Germany). The denaturated sample mix was cooled on ice and transferred to amplification tube strips with lyophilized reaction mixture cakes containing DNA polymerase, random hexamers, salts, buffers, and nucleotides. Whole genome amplification at 30°C proceeded for 2 h, followed by enzyme inactivation at 65°C for 10 min. Samples were purified with the NucleoSpin gDNA Clean-up kit (Macherey-Nagel GmbH & Co., Düren, Germany) according to the manufacturer’s protocol and the amplification was confirmed with agarose gel electrophoresis. DNA concentration was evaluated with the Qubit 2.0 (Life Technologies, Carlsbad, CA, United States), and DNA quality was evaluated with the NanoDrop-1000 spectrophotometer (NanoDrop Technologies Inc., Wilmington, DE, United States). DNA was diluted to a concentration of 10 ng/μl with molecular grade Tris-HCl buffer, pH 8.5 (bioPLUS Buffers and Reagents, Dublin, Ohio, United States). Samples for metagenomic sequencing were sent to Eurofins Genomics Europe Sequencing GmbH, Constance, Germany. Standard metagenomic libraries were prepared at Eurofins Genomics and 10 million read-pairs per sample were produced with Illumina paired-end sequencing (2 × 150 bp). Standard quality control for each library was included.

### Scanning Electron Microscopy

Samples were analyzed with Scanning Electron Microscopy (SEM) after 8 months and at the end of the incubation after 40 months similarly to Rajala et al. (2015b). First samples were fixed with glutaraldehyde (2.5% end concentration), rinsed with a Sörensen phosphate buffer three times, and then dehydrated with an ethanol series as described in Nuppunen-Puputti et al. (2021). The remaining moisture was removed with final dehydration step with hexamethylsilazane (HMDS, Fluka, Switzerland) overnight at the fume hood in an open glass petri dish. Samples were coated with Au/Pd. First time points samples were analyzed at the University of Helsinki, and the second time point samples were analyzed at the Technical Research Centre of Finland with Zeiss ULTRAplus field emission (FE) SEM. The first time point specimens were coated with Au/Pd (10 nm, 208 HR High Resolution Sputter Coater, Cressington Scientific Instruments, Inc., United States) and examined with Hitachi S-4800 FESEM (Japan) operated at 1–5 kV. The second time point specimens were taped to a glass plate using electrically conductive tape to ensure electrical conduction. After the tape was applied, the specimens were sputter-coated with platinum, using Agar Auto Sputter Coater and analyzed with Zeiss ULTRAplus field emission FE (SEM) (Carl Zeiss Microscopy GmbH, Jena, Germany) operated at 7 kV.

### Amplicon Sequence Analysis

Sequence analysis was performed with the DADA2 software (v.1.19.2) (Callahan et al., 2016). Quality was checked with plotQualityProfile followed by trimming to minimum length of 200 with filterAndTrim with options maxN = 0, maxEE = 2, minLen = 200, trunQ = 2, and rm.phix = TRUE. Error rates were estimated with learnErrors. Identical reads were dereplicated followed by inferring of the amplicon sequence variants (ASV) with dada and generation of count table with makeSequenceTable and getSequences. Count tables (RDS-form) from separate iSeq100 runs were merged and checked for chimeric sequences with RemoveBimera*Denovo* consensus method. Bacterial sequences were classified against Silva 138 reference database with DADA2 AssignTaxonomy (Quast et al., 2013), whereas fungal sequences were compared with UNITE v8 (Kõljalg et al., 2013; Nilsson et al., 2019). Contaminant sequences were evaluated based on extraction-wise kit blank controls and PCR mastermix blank controls. For bacteria, ASVs with sequences in control samples were removed from the data, unless the ASVs were represented by over 1,000 sequence reads in the actual samples (DNA), suggesting crossover contamination from, e.g., library preparation. For example, bacterial ASV1 and ASV2 containing under 60 sequence reads in the blank controls were kept in the data when these ASVs were represented by more than 18,000 sequence reads in the actual samples. Most of the removed contaminant ASVs were not present in the actual samples. Contaminant ASVs removed based on the extraction control contained, e.g., genera *Bradyrhizobium, Sphingomonas, Rubellimicrobium, Ralstonia, Herbaspirillum*, and *Methylobacterium-Methylorubrum.* In addition, contamination likely linked to human origin was removed from the data (e.g., *Staphylococcus, Streptococcus*, and Enterobacteriaceae). For the fungal data, all contaminants present in DNA blank controls were removed from the data, except ASV14 that was not detected in DNA samples, but was abundant across the cDNA set. However, this ASV14 was not detected in the cDNA blank control samples. Similarly, despite a maximum of 24 sequence reads in cDNA controls, ASV16 and ASV17 were kept as they were not detected in relevant DNA blank controls and were present in the actual DNA samples (with maximum read counts over 4,000). In the fungal data, contaminant ASVs removed based on blank controls contained, e.g., *Schizophyllum commune, Fusarium keratoplasticum*, and *Candida palmioleophila.* An ASV count and taxonomy tables without contaminant reads were created, used in further analysis, and visualized in phyloseq and AMPVIS2 packages in RStudio (McMurdie and Holmes, 2013; RStudio Team, 2015; Andersen et al., 2018). Principal coordinate analysis (PCoA) from the relative abundance data was performed with Bray–Curtis dissimilarity metric with phyloseq R package, and alpha diversity from raw data with phyloseq estimate_richness (McMurdie and Holmes, 2013).

### Metagenomic Analysis

Metagenomic data preparation was performed in CSC’s Puhti supercomputer research environment (CSC, Finland). Metagenomic raw sequences were first analyzed and merged with SeqPrep (available from https://github.com/jstjohn/SeqPrep). Then, both unmerged (R1 and R2) and merged reads were trimmed with Trimmomatic PE or SE (v. 0.39) with the following parameters: AVGQUAL: 20, HEADCROP: 3, LEADING: 3, TRAILING: 3 and MINLEN: 75 (Bolger et al., 2014). Sequence quality was checked prior to and post trimming with FastQC (v.0.11.8) (Andrews, 2010) and combined to one quality report with multiQC (v.1.11) (Ewels et al., 2016). Samples were co-assembled with MEGAHIT (v.1.2.8) with –min-contig-len 1000 (Li et al., 2015). Assembly statistics were analyzed with Quast (v.5.0.2) (Mikheenko et al., 2016). Sample reads were mapped back to co-assembly with Bowtie2 (v.2.4.4) (Langmead and Salzberg, 2012). Samples were further analyzed in Anvi’o v. 7.0 (Eren et al., 2015, 2021). First anvi’o contigs-database was built with a minimum contig length of 2,000 bp with anvi-gen-contigs-database, and profile databases were built with anvi-profile and samplewise .bam-files. Later merged profile database containing all samples was built with anvi-merge. Automatic binning within Anvi’o was performed with CONCOCT (v.1.1.0) and METABAT2 (v.2.15), and their bins further analyzed and merged with DASTOOL (v.1.13) with a score threshold of 0.4 (Alneberg et al., 2014; Sieber et al., 2018; Kang et al., 2019). Anvi-run-hmms was used to estimate single core copy genes within contig splits. Anvi-scg-taxonomy based on single core copy genes with GTDB was used to estimate taxonomy (Parks et al., 2018, 2020). Bins were manually refined with anvi-refine and checked for differential coverage and sequence composition. Potential outliers were removed. Bins with completeness over 45% and redundancy under 6% were kept for further analysis.

We used additional databases for functional analysis in Anvi’o and the detailed tutorial for metabolic analysis in Anvi’o available at: https://merenlab.org/software/anvio/help/main/programs/anvi-estimate-metabolism/. The metabolic potential of final metagenome assembled genomes (MAGs) was analyzed in Anvi’o for Kegg Orthologs (KO) with run-ncbi-cogs, run-kegg-kofams, and anvi-estimate-metabolism, after first setting up the analysis KEGG database within Anvi’o using setup-ncbi-cogs and setup-kegg-kofam. The Kegg Orthologs (KO) output was called with anvi-estimate metabolism with –kegg-output-modes’ additional option “kofam_hits.” The output was used for carbon cycling and biofilm formation analysis with the Kegg-decoder tool (Graham et al., 2018). Final MAG contig fastas were analyzed with FeGenie and compared against iron metabolism-linked HMM collections (Garber et al., 2020a). In detail, MagicLamp for this with in-built genies LithoGenie, FeGenie, and MnGenie were used (Garber et al., 2020b, available in https://github.com/Arkadiy-Garber/MagicLamp) in order to estimate metabolic potential also for lithotrophic and manganese cycling-linked HMM genes. Modified MagicLamp-included R scripts were used to visualize results as balloon plots based on ggplot2 (Garber et al., 2020a). In addition, we estimated the presence of biofilm formation HMMs with WspGenie (MagicLamp). We used Metabolic v. 4.0 to analyze metabolic traits within MAGs and build pathway cycling maps for carbon, nitrogen, and sulfur (Zhou et al., 2019). METABOLIC-G.pl was run with MAG fasta-files at Puhti (CSC, Finland).

### Data Availability

Project sequences were deposited in the European Nucleotide Archive (ENA) under project accession PRJEB48900.

## Results

### Scanning Electron Microscopy

**FIGURE 1:**
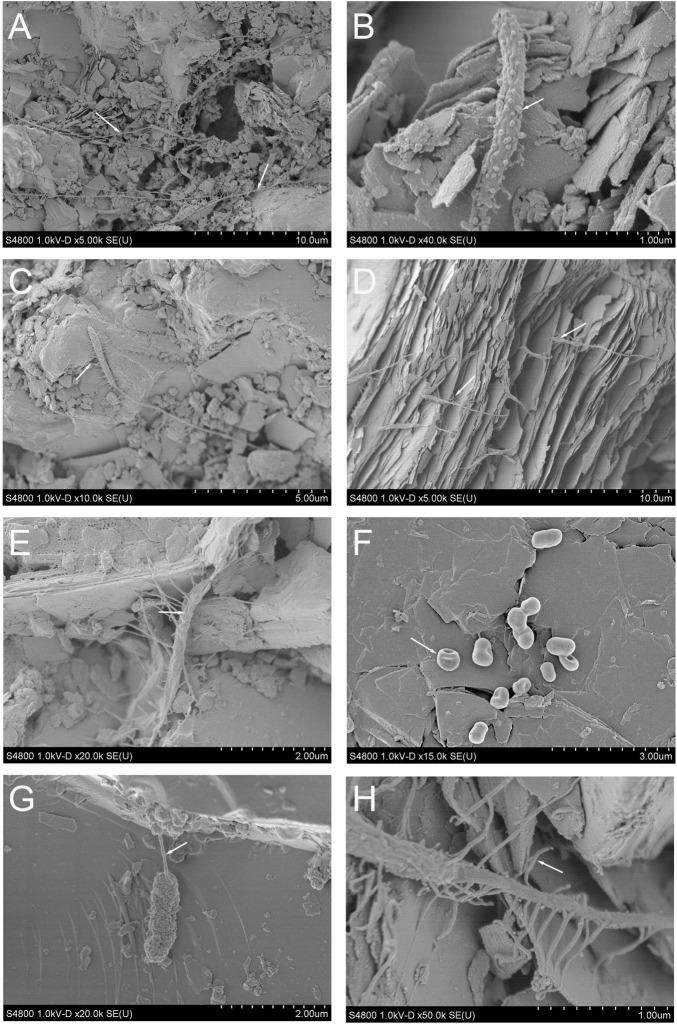
**(A–H)** Scanning electron microscopy images from the mica schist slides after 8 months of incubation. **(A)** Long tubular, attached microbial cell-like structures, **(B)** rod-shaped putative microbial cell with spherical surface attached structures resembling outer membrane vesicles, **(C–E,H)** putative mica schist surface attached microbial cells with hair-like appendices, **(F)** rod-shaped putative microbial cells, **(G)** rod-shaped putative cell with a nanotube-like structure.

**FIGURE 2:**
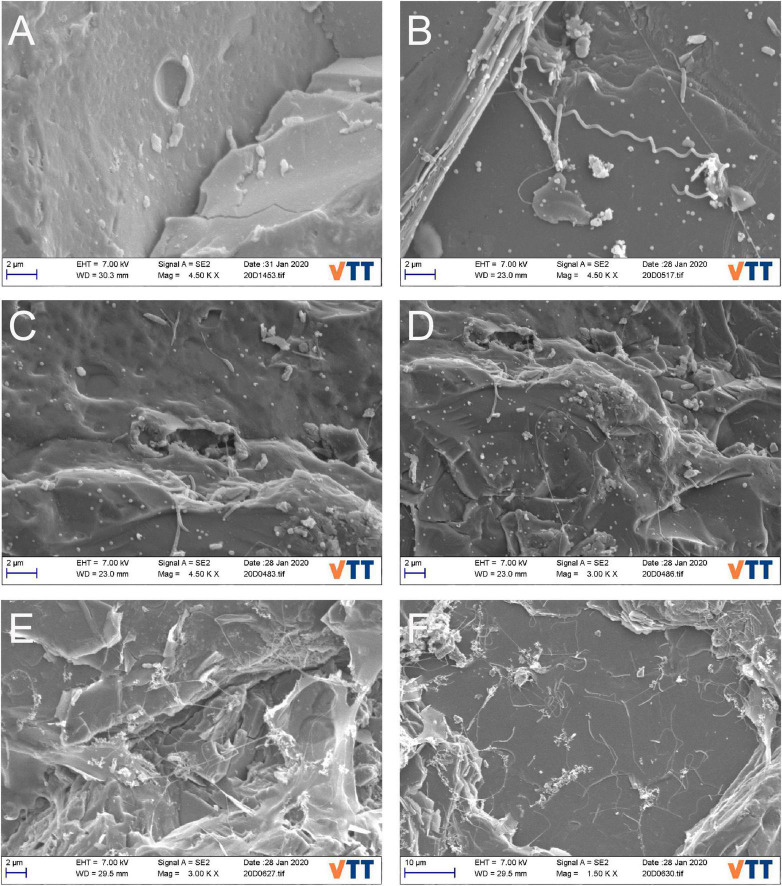
**(A–F)** SEM images for mica schist slide surfaces after 40-month incubation. **(A)** Rod-shaped putative microbial cells and potential pitting, **(B)** potential spirochaeta-like bacterial cell on mica schist surface, **(C,D)** attached putative stalk forming microbial cells and formed pits on surface of mica schist slides, **(E,F)** potential EPS on mica schist surfaces and sessile stalked microbial communities.

The biofilms contained various types of microbial cell-like structures with different morphology types including rods, cocci, spheric, and tubular cells, stalk-forming rods, and spirochaeta-like spiral cells (Figures 1, 2 and Supplementary Figure 1). After 8 months, no dense biofilm was detected and mainly attached single cells were observed. However, possible adhesion mechanisms such as extracellular hair-like appendices and/or potential extracellular slime-like polymers were observed around the cells (Supplementary Figure 1C). Spherical structures resembling membrane-bound vesicles on cell surfaces, potential extracellular polymeric substances (EPS), and fimbriae-like or other nanotubing-resembling structures were observed. Hyphae-like cell tubings expanded over tens to hundreds of micrometers across the mica schist surfaces (Supplementary Figures 1A,B). After 40 months, the mica schist slides had denser biofilm-like biomass with potential EPS (Figures 2E,F) as well as potential microbial pitting on the surface of mica schist (Figures 2A,C,D).

### Microbial Communities

#### Principal Coordinates Analysis for Bacteria

Active bacterial community (identified from cDNA samples) and total bacterial community (identified from DNA samples) showed dissimilarities after 8 months (Supplementary Figure 2). Dissimilarities between glass–surface-attached bacterial communities and planktic water phase communities in the glass-containing microcosms were also detected after 8 months. Most of the mica schist crush microcosm and corresponding water samples clustered closely, indicating similarities in the community structure. As incubation continued, the bacterial communities on glass and mica schist surfaces showed more similarities within their sample type (Supplementary Figure 2). The bacterial communities inhabiting the mica schist slides showed most dissimilarities and formed separate bacterial communities after 40 months of incubation.

#### Bacterial Community Composition

**FIGURE 3:**
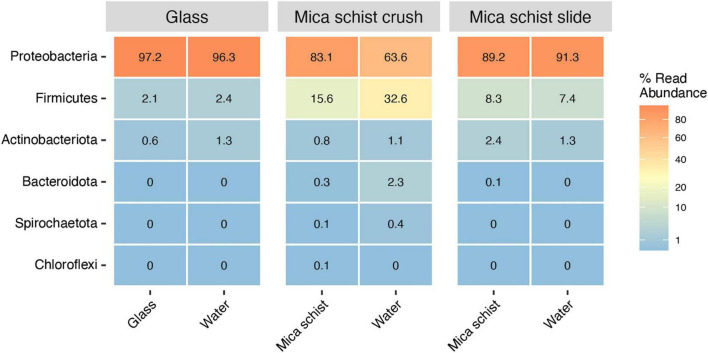
Average relative abundance of bacterial phyla in different sample types (Glass, Mica schist crush and Mica schist slides).

Proteobacteria was the most abundant phylum detected on all surface types, on glass surfaces (97.2%), in MSC (83.1%), and in MSS (89.2%) (Figure 3 and Supplementary Table 2A). The Proteobacteria dominated also in the microcosm water, in glass water (96.3%), in MSC water (63.6%), and in MSS water (91.3%). Firmicutes were enriched especially in mica schist containing microcosms. Actinobacteriota were detected at low relative abundances across all sample types (0.6–2.4%). In addition, Bacteroidota and Spirochaetota were detected in MSC.

**FIGURE 4:**
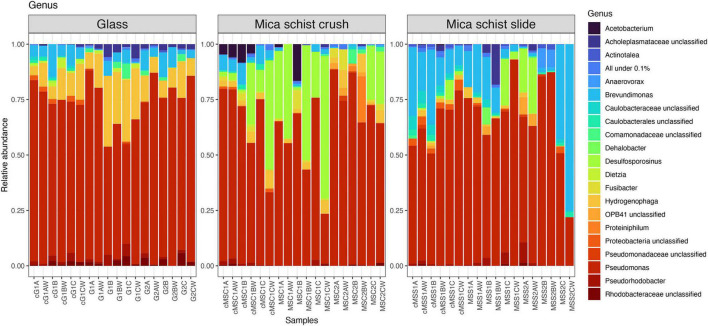
Relative abundances of bacterial communities at genus level in different sample types. All genera below 0.1% mean prevalence were pooled as “All under 0.1%.” The letter “c” in front of the sample names indicates cDNA sample, G = Glass, MSC = Mica schist crush, MSS = Mica schist slide, time point is indicated with 1 or 2, and replicate samples are indicated with A, B, and C for biofilms and AW, BW, and CW for microcosm water phase samples.

*Pseudomonas* was the most prominent genus detected in any type of sample at both time points (Figure 4 and Supplementary Figure 3), although glass, MSC, and MSS had otherwise different community composition patterns. *Hydrogenophaga* had the second highest relative abundance in the glass samples (4.1–31.9%), *Desulfosporosinus* in MSC (0–64.1%), and *Brevundimonas* in MSS (0.3–75%). Unclassified Rhodobacteraceae was more abundant in glass containing microcosm (0–5.5%) than in mica schist microcosms (0–1.3%). Over time, the mica schist surface community structure became more unilateral. After 40 months, all three MSS incubations had developed in different directions, and were dominated by dissimilar bacterial genera in addition to mainly still most abundant *Pseudomonas*. One MSS microcosm had higher relative abundance of *Desulfosporosinus* (19.9% on mica schist), *Pseudorhodobacter* (9.3%), and OPB41 (7.3% on mica schist). The other two MSS replicates hosted either *Anaerovorax* (8.8% on mica schist) or *Brevundimonas* (42.8% on mica schist) as the most prominent genus. All of these genera were detected both from the surface and water of the same microcosm. Similar community simplification was observed in mica schist crush, where *Desulfosporinus* was replaced by, e.g., *Hydrogenophaga* and unclassified *Comamonadaceae* in two replicates, and *Acetobacterium* was not detected anymore. Interestingly, unclassified Caulobacterales and unclassified Caulobacteraceae were detected in the active fraction of the glass and mica schist slide surfaces, and unclassified Caulobacterales also on mica schist crush surfaces, all despite their lower relative abundance in the total bacterial community. These genera were also more prominent in the attached communities compared to the planktic communities of the same microcosm. Other genera detected in the active fraction were, e.g., unclassified Proteobacteria, *Sphaerochaeta*, and *Pseudorhodobacter. Acetobacterium* and *Brevundimonas* were active in mica schist crush after 8 months.

#### Principal Coordinates Analysis for Fungi

Dissimilarities between active fungal community (cDNA) and total fungal community (DNA) after 8 months could be seen as fungal communities separated from each other (Supplementary Figure 4). Planktic or sessile fungal communities in glass and crushed mica schist microcosms were more clearly separated after 40 months, but not after 8 months of incubation. In contrast, fungal community in MSS microcosm was very unilateral after 40 months of incubation. However, the differences were quite small, as shown by the low degree of variance explained by the Axes 1 and 2 (Supplementary Figure 4).

#### Fungal Community Composition

**FIGURE 5:**
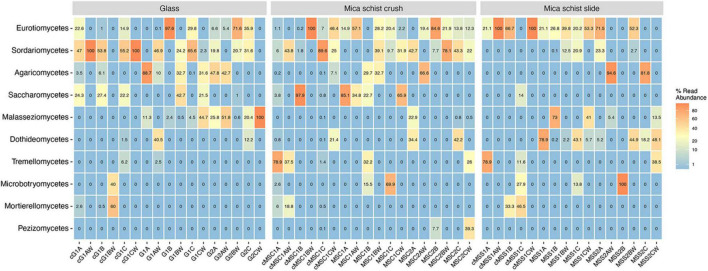
Relative abundance of top ten fungal classes as percentages of reads across sample types. Sample name codes are listed in Table 1.

The fungal phyla detected in mica schist samples from the DNA and cDNA fractions were Ascomycota and Basidiomycota, whereas Mortierellomycota were also detected in the cDNA fractions (Supplementary Table 2B). The most commonly detected fungal classes in MSC were Saccharomycetes (0–85.1%), Eurotiomyces (0–57.1%), and Sordariomyces (0–39.1%) after 8 months (Figure 5, Supplementary Figure 5, and Supplementary Table 2B). Saccharomycetes was not detected after 40 months, when high relative abundances of Sordariomycetes (up to 78.1%) and Eurotiomyces (up to 84.6%) were found. The classes Eurotiomycetales (20.2–53.3%) and Dothideomycetes (0.2–78.9%) were dominant in MSS after 8 months, and Dothideomycetes was still prominent on MSS after 40 months of incubation (0–48.1%). Sordariomycetes was common on glass surfaces as well (up to 65.6%) after 8 months. In addition, glass hosted Agaricomycetes (up to 88.7%) and Malasseziomycetes (up to 44.7%). Sordariomycetes, Eurotiomycetes, and Malasseziomycetes were detected in glass microcosms after 40 months. Sordariomycetes and Eurotiomycetes were the most common fungal classes detected in the active fraction of all microcosms. In addition, Saccharomycetes was frequent in the active fraction of both glass and MSC surfaces and Eurotiomyces in MSS microcosms after 8 months. The most common fungal genera were *Aspergillus* on mica schist surfaces after 8 months and *Malassezia* on glass surfaces (Supplementary Figure 6). *Penicillium* and *Acremonium* were common on MSC after 40 months. *Debaryomyces* and *Fusarium* were the most common genera detected in the active fraction of glass and MSC surfaces. Interestingly, *Penicillium, Fusarium*, and *Debaryomyces* were detected in three active fractions of the glass surface samples, but not in the water samples of the same microcosms. *Penicillium* was also detected in the active fraction of the MSS surfaces.

#### Microbial Community Statistics and Negative Control Samples

The library sizes of the bacterial communities ranged from 116 to 78,230 and from 171 to 3,154 sequence reads per sample at time 1 and time 2, respectively (Supplementary Table 3). The library size for the fungal communities varied between 71 and 121,447 sequence reads at time 1 and between 0 and 17,801 at time 2. The cDNA library sizes ranged between 463 and 2,265 and between 2 and 9,405 sequence reads for the bacteria and fungi, respectively. Archaeal sequences were not detected in the microbial community analysis targeting archaeal 16S rRNA gene region amplification. The number of both observed bacterial ASVs and ASV Chao1 richness varied between 7 and 43 in the mica shist samples over the whole experiment, and between 3 and 23 in the microcosm water (Supplementary Table 4). The Shannon diversity index varied between 0.8 and 2.5 in the mica schist samples and between 0.8 and 2.1 in the water samples from microcosms containing mica schist. The glass samples generally had higher numbers of bacterial ASVs and Chao1 estimated number of ASVs compared to the mica schist samples, but the Shannon diversity indices were similar (Supplementary Table 4). In the cDNA fraction from 8 months, the bacterial ASVs varied between 13 and 23 in the mica schist and between 15 and 28 in the water from mica schist microcosms. In the glass samples and water from glass containing microcosms, the bacterial ASV numbers and estimated ASV numbers were 16–20 in the cDNA fraction. Shannon’s diversity index ranged between 1.4 and 2.3 and between 1.7 and 2.3 in mica schist samples and microcosm water, respectively, and between 1.7 and 2.2 in glass samples and microcosm water with glass.

The number of fungal ASVs and Chao1 estimated number of ASVs on mica schist varied between 1 and 12 and between 2 and 12, respectively, whereas the values in mica schist microcosm water were 2–14 and 2–15, respectively (Supplementary Table 5). The number of fungal ASVs and Chao1 estimated ASVs in glass and glass microcosm samples were 3–19 and 6–25, respectively. The fungal Shannon index for mica schist and microcosm water with mica schist were 0.5–2.2 and 0.2–1.7, respectively. In glass and glass microcosm water, the respective values were 0.8–2.3 and 0–2.3, respectively. In the cDNA fraction, the MSC had the highest number of observed and estimated fungal ASVs, 5–25, whereas the MSS, MSC, and MSS water had between 1 and 6 fungal ASVs. The Shannon index was between 0 and 2 in all mica schist and mica schist water samples, with the highest index in the MSC. The glass surfaces had higher numbers of fungal ASVs from the cDNA fraction compared to the water from the glass microcosms and had also high diversity indices of up to 1.9, compared to 0–1.1 on the glass surfaces.

Negative incubation control samples for glass, MSC, and MSS all contained low amounts of bacterial sequences in the moderated sequence data, whereas second time point DNA and cDNA samples contained more sequences in control samples equimolarly pooled for the amplicon sequencing libraries (Supplementary Table 3). The number of bacterial sequences in the 40-month incubations was higher, but likely represented crossover from library preparation. SEM analysis showed that mica schist slide samples from the negative controls did not contain any microbial cells in either time points.

### Metagenomics of the Mica Schist Surface Microbial Communities

#### Quality Filtering and Assembly Statistics

After quality filtering, the average sequence length varied between 151 and 225 bp and GC% varied between 44 and 57%. No adapter contaminated or over-represented sequences passed the quality filtering steps. Metagenomic reads from 12 whole genome amplified mica schist surface DNA samples were co-assembled and genome fraction in final contigs represented 54% of the sequences (Supplementary Table 6). The largest contig was 126,957 bp and total assembly length was 157,850,493 bp.

#### Metagenomic Binning

Metagenomic co-assembly with MEGAHIT produced 66 bins identified by CONCOCT and 81 bins identified by METABAT2, but after analysis with DASTOOL, the dataset contained 22 merged bins. The final merged DASTOOL dataset represented in total 58.46% of the nucleotides in the contig and profile databases in Anvi’o. After manual refinement of bins in Anvi’o, the dataset contained 21 metagenomic assembled genomes (MAGs), out of which eight were excellent quality (> 90% completion, < 5% redundancy), four MAGs were 80–90% complete, eight MAGs were medium quality (> 50% completion, < 5% redundancy, except for MAG17 with 5.63% redundancy), and one was a low-quality draft MAG (45% complete, < 5% redundant) (Table 2). The completeness of the MAGs was estimated with anvi-hmms.

**TABLE 2:** Metagenome assembled genomes.

MAG ID	Total length (bp)	Number of contigs	N50	GC content	Completeness	Redundancy
OKU01	3,526,166	147	38,422	43.25	100.00	1.41
OKU02	2,418,070	59	60,346	38.36	98.59	2.82
OKU03	4,683,040	162	48,463	41.75	95.77	1.41
OKU04	3,177,924	63	80,327	47.19	94.37	1.41
OKU05	4,483,115	181	42,831	43.02	94.37	1.41
OKU06	1,759,517	105	25,694	31.13	91.55	2.82
OKU07	1,592,376	133	18,186	35.94	90.14	1.41
OKU08	1,929,415	213	16,679	63.73	88.73	1.41
OKU09	3,672,386	94	62,676	45.87	90.14	2.82
OKU10	3,552,447	285	20,406	51.28	87.32	2.82
OKU11	2,833,120	292	14,888	38.08	87.32	2.82
OKU12	2,702,769	449	7,899	67.23	81.69	1.41
OKU13	3,575,300	50	117,471	43.97	76.06	0.00
OKU14	1,216,298	161	12,631	63.36	73.24	0.00
OKU15	1,317654	192	10,078	58.95	70.42	0.00
OKU16	3,050,115	578	6,377	58.11	70.42	0.00
OKU17	1,834,147	349	6,435	65.94	74.65	5.63
OKU18	2,077,283	222	14,530	39.22	66.20	0.00
OKU19	1,936,026	211	13,841	33.05	63.38	2.82
OKU20	4,675,564	421	16,632	50.07	52.11	1.41
OKU21	1,565,446	70	25,360	39.75	45.07	0.00

**MAG ID**	**Kingdom**	**Phylum**	**Class**	**Order**	**Family**	**Genus**

OKU01	Bacteria	Firmicutes	Clostridia	Eubacteriales	Eubacteriaceae	Acetobacterium
OKU02	Bacteria	Firmicutes	Clostridia	Peptostreptococcales	Fusibacteraceae	UBA5201
OKU03	Bacteria	Firmicutes	Desulfitobacteriia	Desulfitobacteriales	Desulfitobacteriaceae	Desulfosporosinus
OKU04	Bacteria	Bacteroidota	Bacteroidia	Bacteroidales	Dysgonomonadaceae	Proteiniphilum
OKU05	Bacteria	Firmicutes	Desulfitobacteriia	Desulfitobacteriales	Desulfitobacteriaceae	Desulfosporosinus
OKU06	Bacteria	Firmicutes	Bacilli	Acholeplasmatales	Acholeplasmataceae	MZ-XQ
OKU07	Bacteria	Firmicutes	Bacilli	Acholeplasmatales	UBA5453	UBA6235
OKU08	Bacteria	Actinobacteriota	Coriobacteriia	OPB41	UBA2279	UBA2286
OKU09	Bacteria	Chloroflexota	Anaerolineae	Anaerolineales	Anaerolineaceae	UBA6107
OKU10	Bacteria	Spirochaetota	Spirochaetia	Sphaerochaetales	Sphaerochaetaceae	UBA8525
OKU11	Bacteria	Firmicutes	Clostridia	Tissierellales	Dethiosulfatibacteraceae	38–11
OKU12	Bacteria	Proteobacteria	Alphaproteobacteria	Caulobacterales	Caulobacteraceae	Brevundimonas
OKU13	Bacteria	Firmicutes	Clostridia	Peptostreptococcales	Anaerovoracaceae	UBA7709
OKU14	Bacteria	Actinobacteriota	Coriobacteriia	Coriobacteriia	Coriobacteriia	Coriobacteriia
OKU15	Bacteria	Proteobacteria	Gammaproteobacteria	Pseudomonadales	Pseudomonadaceae	Pseudomonas
OKU16	Bacteria	Proteobacteria	Gammaproteobacteria	Pseudomonadales	Pseudomonadaceae	Pseudomonas
OKU17	Bacteria	Proteobacteria	Gammaproteobacteria	Burkholderiales	Burkholderiaceae	Serpentinomonas
OKU18	Bacteria	Firmicutes	Desulfitobacteriia	Desulfitobacteriales	Syntrophobotulaceae	Gracilibacter
OKU19	Bacteria	Firmicutes	Bacilli	Acholeplasmatales	Acholeplasmataceae	UBA2284
OKU20	Bacteria	Firmicutes	Bacilli	Paenibacillales	Paenibacillaceae	Paenibacillus
OKU21	Bacteria	Firmicutes	Bacilli	Erysipelotrichales	Erysipelotrichaceae	UBA2227

*MAG IDs, total length (bp), number of contigs, N50, GC content as percentage (GC%), MAG completeness% (Comp.), and redundancy% (Red.) values. In addition, the putative taxonomy of the MAGs assigned in Anvi’o is based on the GTDB taxonomy. All MAGs affiliated with Bacteria.*

#### Biofilm Formation and Motility

**FIGURE 6:**
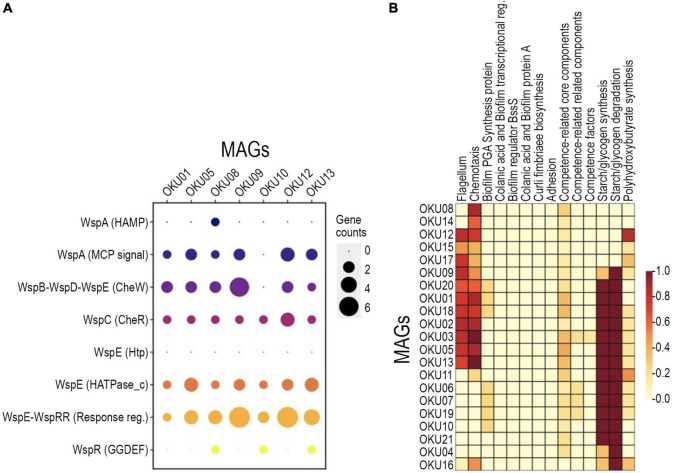
Detected biofilm formation genes across MAGs with detected annotation matches in WspGenie analysis **(A)** and biofilm formation genes in KeggDecoder analysis **(B)**. Gene counts were compared against contig gene count in WspGenie.

A variety of genes linked to biofilm formation, i.e., *Wsp* genes, were detected in seven MAGs (Figure 6A and Supplementary Table 7C). In contrast, no WspF genes linked to hindering of the initiation of biofilm formation were detected in any MAGs. Biofilm formation-linked genes such as putative diguanylate cyclase (c-diGMP) synthase gene *Wsp*R (GGDEF domain) were detected in OKU08, OKU10, and OKU13 (Figure 6A). WspC genes (CheR domain), and WspB-WspD-WspE (CheW-like domain) were detected in seven and six MAGs, respectively (Figure 6A). In addition, nine MAGs had genes for producing flagella, and 15 MAGs had genes for chemotaxis (Figure 6B). Putative genes for the poly-beta-1,6-N-acetyl-D-glucosamine (PGA) synthesis proteins were detected, and genes for the starch/glycogen formation and degradation were common across MAGs (Figure 6B and Supplementary Tables 7, 9). All MAGs except OKU 16 had core competence genes.

#### Diverse Metabolic Patterns in Epilithic Microbial Communities

**FIGURE 7:**
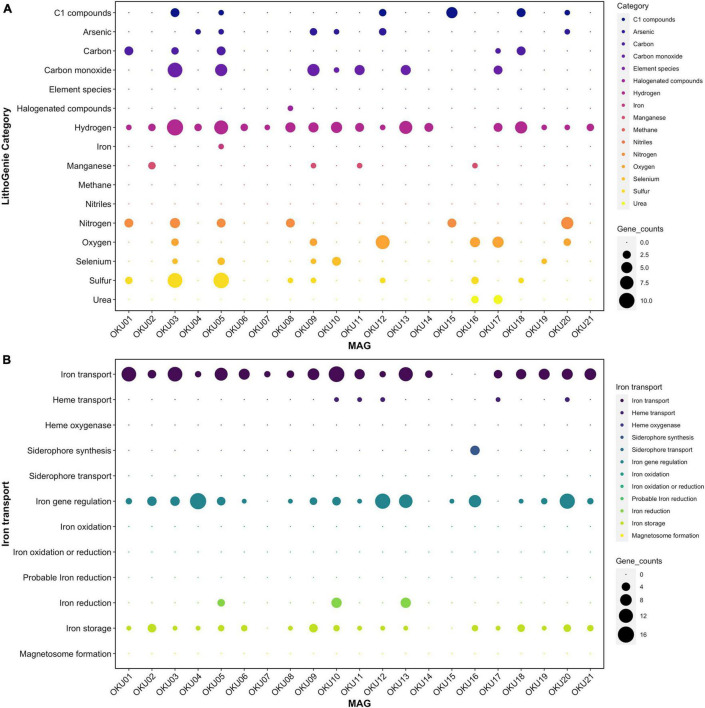
Metabolic potential in different MAGs analyzed with LithoGenie **(A)** and FeGenie **(B)**. MAG contig open reading frames (ORFs) were first analyzed with Prodigal from contig nucleic acid sequences whereafter amino acid sequences were compared against LithoGenie and FeGenie HMM gene databases. Gene counts were calculated separately for each Genie visualization.

Metabolic traits were analyzed with several approaches and predicted genes and their matches with homologous sequences may not always represent identical functionality. The program-wise detection thresholds for hidden Markov model (HMM)-based gene collection matches and pathway module completeness were used. Diverse metabolic patterns were detected across epilithic microbial MAGs, e.g., in *Desulfosporosinus* MAGs (OKU03 and OKU05) (Figure 7 and Supplementary Tables 7A,B). These known sulfate reducers showed also potential for carbon monoxide and nitrogen cycling, and ability to utilize C1 compounds. Hydrogen cycling genes were ubiquitous across all MAGS, lacking only from *Pseudomonas* MAGs (OKU15 and OKU16). Interestingly, arsenate and selenate reduction genes were quite common in the epilithic MAGs.

#### Carbon Cycling

**FIGURE 8:**
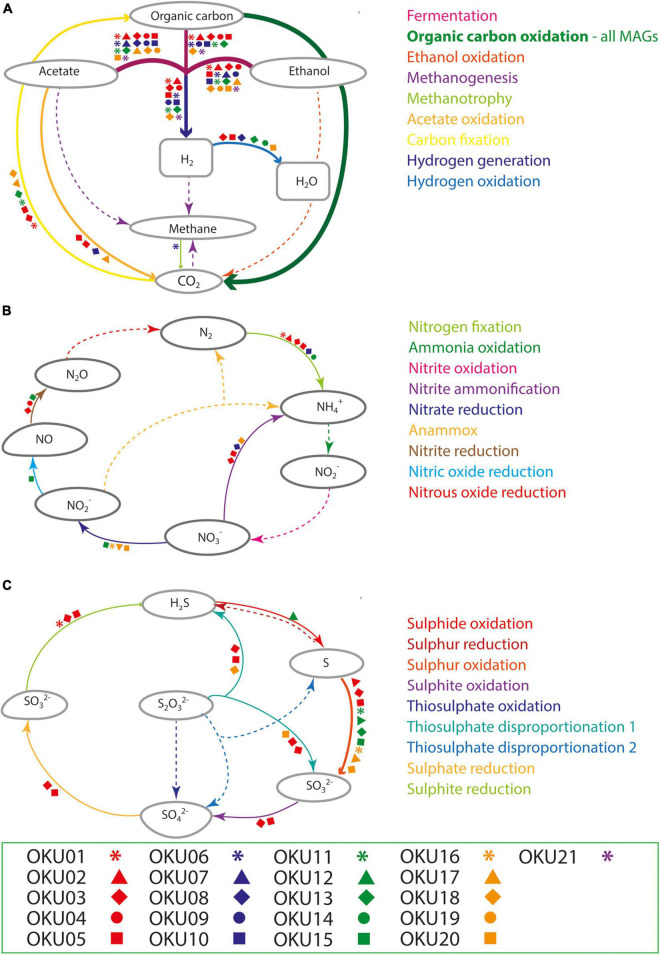
Compilation of carbon **(A)**, nitrogen **(B)**, and sulfur **(C)** cycling pathways across mica schist MAGs according to METABOLIC analysis. Detected pathways are marked with arrows, whereas missing pathways are marked with dashed arrows. Different MAGs linked to the detected pathways are marked with the symbols according to the legend.

**FIGURE 9:**
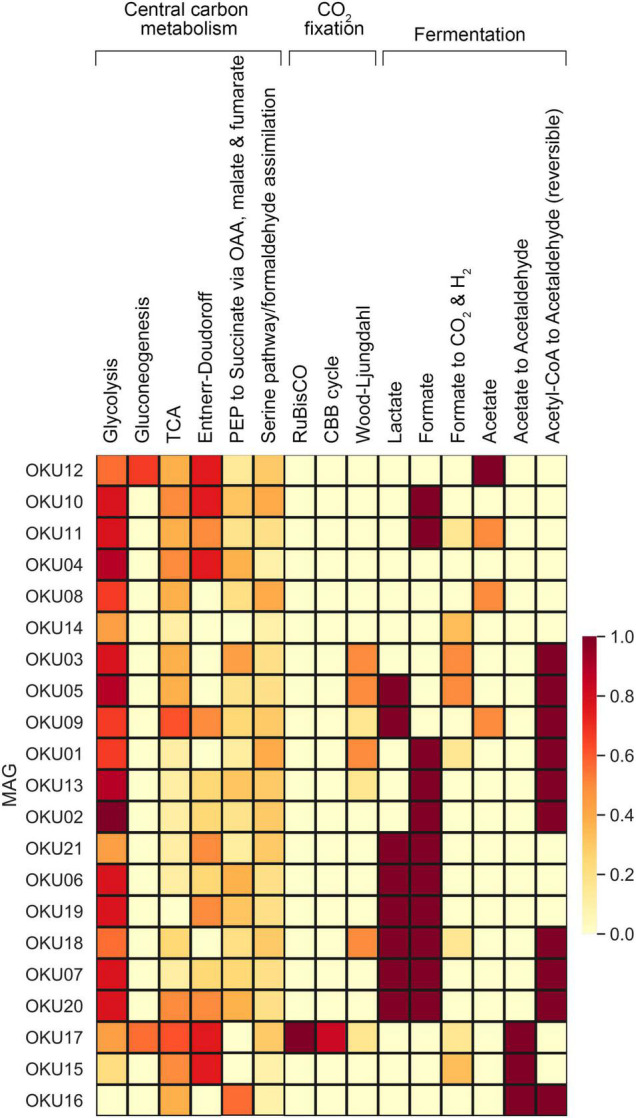
Central carbon metabolism, carbon fixation pathways, and mixed acid cycle completeness estimated with Keggdecoder. Dark red color indicates full pathway, orange partially detected pathways, and light-yellow color indicates a missing pathway.

Multiple carbon cycling pathways were detected across the epilithic MAGs, including fermentation, organic carbon oxidation, carbon fixation, acetate oxidation, and methanotrophy (Figure 8). In addition, carbon cycling was linked to generation and oxidation of hydrogen. Genes linked to fermentation and acetyl-CoA formation (acdA/ack/pta) were detected in 17 MAGs, whereas the acetate oxidation gene (acs) was detected in only four MAGs (Figure 8A and Supplementary Table 9). Potential to form acetyl-CoA and formate through fermentation of pyruvate (pflD) was found in 10 other MAGs. The complete TCA cycle was not detected in any MAG and no genes for the rTCA cycle were detected (Figure 9 and Supplementary Tables 7, 9). Glycolysis genes were very common in all rock surface MAGs, yet the full pathway was found only in keggdecoder analysis (OKU2) (Figure 9 and Supplementary Tables 8, 9). Partial gluconeogenesis was detected in only two MAGs (OKU12 and OKU17). Five MAGs contained genes for carbon cycling linked mainly to carbon fixation *via* the Wood-Ljungdahl pathway (Figure 7A and Supplementary Tables 7–9). Interestingly, twelve MAGs contained genes for complex carbon degradation, e.g., chitinase and hexoaminidase.

##### Carbon Fixation and CO Oxidation

Carbon fixation pathway genes for RuBisCO and the partial CBB cycle was found in OKU17 (Figure 9 and Supplementary Table 9). Partial Wood-Ljungdahl pathway (c*dh*D, *cdh*E, and *coo*S) was identified in four MAGs (OKU01, OKU03, OKU05, and OKU18), which also showed complete acetogenesis pathways linked to the Wood-Ljungdahl pathway (Supplementary Table 8). In addition, OKU13 also contained the *coo*S gene for the catalytic subunit of the anaerobic carbon monoxide dehydrogenase (Supplementary Table 9). Aerobic carbon monoxide oxidation genes (*cox*S, *cox*M, and *cox*L) were detected in various MAGs (Supplementary Table 7). Full sets of these genes were detected in OKU09 and OKU17, which lacked anaerobic carbon monoxide dehydrogenase genes (CODH) for the full Wood-Ljungdahl pathway (Figure 7).

##### C1 Cycling

C1 cycling genes were detected in six MAGs (Figure 7A). Formaldehyde oxidation genes for production of glutathione or formate (*fgh*A and *frm*A) were found in OKU12, whereas formate oxidation genes linked to formation of CO_2_ and NADH or reduced coenzyme F420 were found in seven MAGs (Supplementary Table 9). Methanol oxidation (*mxa*F) potential was found in OKU15 and only the *mmo*B gene encoding the methane oxidation regulatory protein B of the soluble methane monooxygenase indicating any oxidation of methane to methanol was detected in OKU06 affiliating with Acholeplasmataceae. A variety of genes encoding enzymes included in the methanogenesis pathways were found across MAGs (Figure 9 and Supplementary Tables 8, 9). However, the final enzyme (Coenzyme M reduction to methane) needed in order to produce methane was missing from all genomes as expected with bacterial MAGs. Dimethylamine/trimethylamine dehydrogenase was found in putative Pseudomonas MAG16 (Supplementary Table 8).

#### Nitrogen Cycling

Nitrogen cycling genes were found in ten MAGs (Figures 7A, 8B and Supplementary Figure 7) and were involved in nitrogen fixation, nitrate and nitrite reduction, nitric oxide reduction, and nitrite ammonification (Figure 8B). None of the MAGs had genes for any oxidative pathways in nitrogen cycle such as ammonia oxidation. Genes for N_2_ fixation to ammonia (*nif*D, *nif*H, and *nif*K) were found in six MAGs (Figure 8B and Supplementary Table 9). Genes for nitrate reduction to nitrite (*nar*G and *nar*H) were found in OKU17 and OKU20, whereas nitrite reduction genes (*nap*A and/or *nap*B) were found in the *Pseudomonas* MAGs (OKU15 and OKU16). Nitrite reduction to ammonia (ammonification) genes (*nir*B and *nir*D) were found in OKU20, and genes for nitrite reduction to ammonia (*nrfH* and/or *nrf*A) were found in the *Desulfosporosinus* MAGs (OKU03 and OKU05) and in OKU08. Nitric oxide reduction genes (*nor*BC) were found in OKU03, OKU04, and OKU15. *Pseudomonas* MAGs also contained urease genes (*ure*A, *ure*B, and *ure*C) linked to production of ammonia (Figure 7A and Supplementary Table 9).

#### Sulfur Cycling (Including Dimethylsulfoxide)

A wide variety of sulfur cycling genes were detected in mica schist community MAGs, such as genes involved in dissimilatory sulfate, sulfite and sulfur reduction, sulfur and sulfite oxidation, as well as thiosulfate disproportionation to sulfite and hydrogen sulfide (Figure 8C). Pathways for thiosulfate oxidation or disproportionation to sulfate and elemental sulfur were not detected (Figure 8C). Only the *Desulfosporosinus* MAGs (OKU03 and OKU05) contained genes for dissimilatory sulfate reduction, i.e., adenylylsulfate reductase and sulfate adenylyltransferase (*apr*A, *apr*B, and sat) and dissimilatory sulfite reductase genes (*dsr*ABCD and *dsr*JKMOP) (Figure 8C and Supplementary Table 9). Assimilatory sulfur cycling genes for sulfur oxidation (sdo) and DMSO/DMS/methanethiol transformations (*ddh*A and *dmo*B) were detected in all MAGs, except OKU11 (Dethiosulfatibacteraceae). Anaerobic sulfite reduction genes (*asr*ABC) were solely detected in OKU01. Thiosulfate disproportionation genes (*phs*A) were found in *Desulfosporosinus* and *Gracilibacter* affiliating MAGs. Sulfide oxidation linked sulfide-quinone reductase gene (sgr) was found in OKU12 (*Brevundimonas*). This gene is linked to formation of polysulfides. No genes for sulfur reduction or production of sulfides were found. A variety of dimethylsulfoxide (DMSO) metabolism linked genes were detected across MAGs (Supplementary Table 9). For example, DMSO, DMS, and methanethiol could be formed in these reactions. Only *Pseudomonas* (OKU16) contained a gene (*sfn*G) for DMSO/methanesulfonate transformation.

#### Manganese, Arsenate and Selenate Cycling

Manganese oxidase genes were found in four MAGs (Figure 7A). The *mco*A gene was found in MAGs OKU02 and OKU11, whereas the *mop*A gene was found in MAGs OKU09 and OKU16 (Supplementary Table 7A). In addition, manganese transport genes, such as ABC transporters and iron/manganese superoxide dismutase genes, as well as manganese binding enzyme genes such as manganese containing catalases were common (Supplementary Figure 8 and Supplementary Table 7D). Genes for arsenate reduction with thioredoxin (*ars*C2) were ubiquitous in rock surface MAGs and found in 16 out of 21 MAGs and a different gene for arsenate reduction with glutaredoxin was present in two MAGs (Figure 7A and Supplementary Tables 7, 9). Genes for selenate reduction to elemental selenium (*ygf*M, *xdh*D, and *ygf*K) were found in rock surface MAGs and a full set with all these genes was found in OKU10 (Figure 7A and Supplementary Tables 7, 9).

#### Iron and Hydrogen Cycling

Most of the detected genes for iron utilization were related to the acquisition of iron. These genes covered transport and/or siderophores, iron gene regulation, and storage of iron (Figure 7B). Putative iron reduction genes were detected in MAGs OKU05, OKU10, and OKU13 (Figure 7B). Iron reduction genes detected in OKU05 were DFE_0448, DFE_0449, and DFE_0451 in addition to *Dmk*B. In contrast, a different set of genes for iron reduction including Ndh2, Dmk(AB), Eet(AB), and FmnB were detected in both OKU10 and OKU13, whereas PplA was also found in OKU10. Iron oxidation genes were not found. Detected hydrogenases belonged to the FeFe Hydrogenase groups A and B1B3 and to Hydrogenase groups 1, 3b, and 4 (Figure 7A and Supplementary Tables 7, 9). All these hydrogenase genes were detected in the sulfate reducer MAG OKU05, and the other SRB MAG, OKU03, was missing only in Hydrogenase group 3b. H_2_ evolving NiFe-hydrogenases (Group 4a–g) were found in the SRB MAGs, in addition to OKU13 (Supplementary Table 9). NADP-reducing hydrogenase and NiFe hydrogenase Hyd-1 genes were common in rock surface MAGs (Supplementary Tables 7, 9).

## Discussion

### Main Epilithic Bacterial Communities in Outokumpu Deep Subsurface

Microorganisms have been found to inhabit deep crystalline bedrock to several kilometer depths. In general, microbial communities have been studied in fluid samples and only recently have studies focused on mineral surface biofilm functionality (Casar et al., 2021a,b; Nuppunen-Puputti et al., 2021; Sanz et al., 2021). Biofilms have been studied from drill core fractures, flow cells, and *in situ* biofilm traps (Pedersen et al., 1996; Jägevall et al., 2011; Wu et al., 2017; Escudero et al., 2018; Casar et al., 2021b; Nuppunen-Puputti et al., 2021). In our study, we incubated mica schist containing deep groundwater microcosms for up to 40 months to study the formation of biofilm on rock surfaces. We show that the sessile rock surface microbial communities have a wide morphologic variety and metabolic potential, giving new insight into lesser-known deep subsurface biofilm communities. We detected differences in microbial communities depending on the sample type (MSS or MSC). Rough surfaces potentially advance microbial attachment, while enhanced nutrient availability in crushed mica schist samples likely supports different genera compared to the mica slides. Carbon sources in deep oligotrophic subsurface are limited and especially carbon cycling strategies in Outokumpu deep groundwater have been addressed with multiple approaches (Nyyssönen et al., 2014; Purkamo et al., 2014, 2017; Rajala et al., 2015a; Bomberg et al., 2017; Nuppunen-Puputti et al., 2018). Here, we demonstrate that rock surface communities have multiple carbon cycling patterns ranging from core carbon metabolism to carbon monoxide oxidation, utilization of C1 compounds, carbon fixation, and complex organic matter degradation through various enzymes like chitinases and pullulanases.

*Pseudomonas* was the most dominant bacterial genus detected in both mica schist crush and slides at both 8 and 40 months. *Pseudomonas* has been identified as one of the Outokumpu deep subsurface keystone species, and part of the core bacterial community in fracture fluid at 500 m depth with a relative abundance of 6% (Purkamo et al., 2016). *Pseudomonas* has been shown to assimilate carbonates at 180 m depth in the Outokumpu groundwater (Bomberg et al., 2017), and in deeper parts of the Outokumpu deep drill hole, *Pseudomonas* has been shown to participate on the cycling of acetate (Nuppunen-Puputti et al., 2018). Here, interestingly, rock surface *Pseudomonas* MAGs OKU15 and OKU16 did not have any genes for carbon fixation, but instead showed a high metabolic variability as one genome contained genes for nitrate and C1 compound cycling, and the other for sulfur cycling and manganese oxidation. The C1 compound utilization identified from *Pseudomonas* (OKU15) included methanol and formate oxidation linked to producing rather than consuming CO_2_ in deep subsurface. *Pseudomonas* OKU15 hosted pathways for ethanol fermentation through acetate and acetaldehyde.

Other major epilithic bacterial genera detected on mica schist were *Desulfosporosinus, Hydrogenophaga*, and *Brevundimonas. Desulfosporosinus* have previously been detected from Outokumpu deep subsurface groundwater (Itävaara et al., 2011; Bomberg et al., 2017). *Desulfosporosinus* sp. has been enriched from cultures below 400 m depth in the IPB and the genomic analysis of the isolate revealed potential for CO_2_ and N_2_ production (Sanz et al., 2021). The OKU03 and OKU05 deep bedrock epilithic *Desulfosporosinus* MAGs contained a gene pool that could enable them to switch metabolic strategies when needed. The *Desulfosporosinus* MAGs have “the tools” for metabolic cycling linked to sulfur, nitrogen, carbon, hydrogen, arsenic, and selenium. Acetogenesis modules were detected in four MAGs. Two of these matched with *Desulfosporosinus*, which is a known sulfate reducing genus and known to produce acetate either from H_2_ and CO_2_ or *via* an electrosynthetic pathway (Agostino et al., 2020). Acetate is an important intermediate compound in deep subsurface providing substrates for methanogenesis (Lever, 2012; Purkamo et al., 2017; Miettinen et al., 2018).

*Hydrogenophaga* was typical in the glass surface microbial communities detected by amplicon sequencing and was one of the major epilithic genera. *Serpentinomonas* is closely related to *Hydrogenophaga* (Suzuki et al., 2014), which could explain why binning of *Hydrogenophaga-*related sequences was not successful. Suzuki et al. (2014) described a new *Serpentinomonas* genus and three *Serpentinomonas* strains utilizing carbonates and hydrogen autotrophically (Suzuki et al., 2014). They also affiliated these new *Serpentinomonas* strains with previous Outokumpu deep drill hole *Hydrogenophaga* short read OTUs (Itävaara et al., 2011; Suzuki et al., 2014). *Serpentinomonas* has been shown to grow on acetate (Suzuki et al., 2014), which is in agreement with our data, showing that OKU17 (*Serpentinomonas*) contained genes for acetate oxidation (Figure 8A and Supplementary Table 9).

*Brevundimonas* (OKU12) and other Caulobacteraceae genera were common especially on MSS. This could indicate a competitive advantage in the initiation phase of the biofilm gained from their stalk formation ability advancing attachment and contact with the rock surface. *Brevundimonas* was one of the active genera, as detected from cDNA, in all sample types after 8 months of incubation. *Brevundimonas* OKU12 showed genetic potential for both sulfide and sulfur oxidation. In addition, genes for formaldehyde oxidation and oxidative phosphorylation were detected. *Brevundimonas* has been shown to have sulfide oxidation genes in another Finnish deep subsurface location in Olkiluoto (Bell et al., 2020). These findings clearly link this genus to the deep subsurface sulfur cycle in both groundwater and rock surface communities. In addition, *Brevundimonas* (OKU12) contained genes encoding enzymes for complex carbon compound degradation. Putative *Caulobacteraceae-*like bacterial cells with elongated stalks were observed on mineral surfaces at both time points in SEM analysis (Figures 1C, 2C,E,F and Supplementary Figure 1B). Previously, *Caulobacter* have been shown to enhance their stalk formation under phosphate limitation (Schmidt and Stanier, 1966; Gonin et al., 2000). Thus, phosphate limitation could cause the elongated morphology of the *Brevundimonas-*like long stalks on deep subsurface mineral surfaces (Supplementary Figure 1B). Bryce et al. (2016) suggested phosphate sorption into mineral surfaces as a trigger for microbial phosphate limitation response observed in rock dwelling microbe proteome (Bryce et al., 2016). The *Brevundimonas* (family Caulobacteraceae) MAG (OKU12) contained ProR-PhoB genes linked to phosphate starvation response identified in *Caulobacter* species (Gonin et al., 2000). In *Caulobacter* sp., the elongation of cellular structures enhances nutrient uptake capability through larger surface/volume ratio (Gonin et al., 2000). However, here we describe a closed microcosm enrichment system, and the deep subsurface *in situ* fluid flow and gas exchange could affect this phenomenon. *Brevundimonas* affiliating phylotypes were identified in previous enrichment studies where deep crystalline bedrock fluids were enriched with organic acid amendments (Fukuda et al., 2010; Purkamo et al., 2017).

### Metabolic Traits of Low Abundance Phyla Attached on Mica Schist

Actinobacteria (OKU08 and OKU14), Spirochaeta (OKU10), and Chloroflexota (OKU09) represented minor phyla of the MSC microbial communities identified by the amplicon sequencing. Actinobacterial Coriobacteriia MAGs had genes for nitrogen fixation to ammonia and OKU08 also for nitrite ammonification. Both of these pathways produce ammonia, which make them exceptionally important as replenishers of the available nitrogen pool in oligotrophic deep subsurface. Coriobacteriia MAGs differed in their putative carbon metabolism as OKU08 had genes for potential utilization of acetate and fermentation, whereas OKU14 had only C1 cycling genes for formate oxidation. Spirochaeta and Chloroflexota hosted genes for complex carbon degradation, fermentation, hydrogenases, and arsenate and selenate reduction. In both Chloroflexota and Spirochaeta MAGs, carbon metabolism centered on fermentation and organic carbon oxidation, thus producing hydrogen and CO_2_. In addition, Chloroflexota had a set of *cox* genes for carbon monoxide oxidation. None of these groups were linked to the dissimilatory sulfur cycle, but Spirochaeta and Chloroflexota MAGs had genes for DMS–DMSO transformation. Chloroflexota belonging to the class Anaerolinea have also been detected in deep subsurface environments such as in the Western Siberia (Kadnikov et al., 2018), where Anaerolinea were shown by metagenomics to rely on fermentation as their major energy acquisition strategy in deep sedimentary subsurface (Kadnikov et al., 2020).

We obtained three Acholeplasmatales MAGs, affiliating with genera MZ-XQ (OKU06), UBA6235 (OKU07), and UBA2286 (OKU19). Acholeplasmas (Mollicutes) have been shown to be part of the continental deep subsurface, and in Outokumpu, they have been detected in groundwater microbial communities at different sampling depths (Purkamo et al., 2013, 2016). Acholeplasma in Tenericutes phylum are microbes lacking peptidoglycan cell walls (Wang et al., 2020). Most Acholeplasmas are endosymbionts, but also free-living environmental deep-sea isolates have been found (Wang et al., 2020). Originally, the nearest affiliation to OKU06, MZ-XQ strain, was isolated from deep oceanic subsurface coalbed bioreactor enrichment (Imachi et al., 2019). As expected for assumed endosymbionts, the metabolic range in the OKU MAGs was relatively low. All three Acholeplasma MAGs have fermentation-linked genes for L-lactate dehydrogenase, acetate kinase, and formate C-acyltransferase, and a nearly full set of genes for the glycolysis pathway. OKU06 contained the *mmo*B gene involved in the regulation of methane oxidation to methanol and two different groups of FeFe hydrogenases. All Acholeplasma MAGs contained genes for arsenate reduction and OKU19 also for selenate reduction. Acholeplasma MAGs hosted a wide variety of transport-related genes. In addition, these Acholeplasmas had complex carbon degradation-linked genes for alpha-amylases, beta-glucosidases, pullulanases, and chitin degradation. Free-living Acholeplasmas have been shown to participate in degradation and/or uptake of microbial biomass including DNA (Hanajima et al., 2015). Dead biomass has been shown to be efficiently degraded in deep groundwaters and further used for synthesis of new biomass (Lopez-Fernandez et al., 2018). In the oligotrophic deep biosphere, everything is recycled, and with this enzyme pool for degrading complex carbon, the saprotrophic Acholeplasmas are likely able to degrade and use dead bacterial and fungal biomass. Chitinases were commonly encountered across also other MAGs, suggesting that the bacteria could dissolve dead fungal biomass in deep subsurface.

Overall heterotrophic metabolic traits are more common in epilithic community MAGs than autotrophic metabolic strategies (Figure 8). Similarly, previous studies on planktic deep groundwater communities from several depths in Outokumpu indicated that the deep biosphere rather functioned on heterotrophy than autotrophy (Nyyssönen et al., 2014; Purkamo et al., 2014; Bomberg et al., 2017).

### Epilithic Community Development

We compared microbial community enrichment strategies on rock surfaces and community structures developed in the microcosms to our previous *in situ* incubation (Nuppunen-Puputti et al., 2021). As in the present microcosm experiment, Proteobacteria and Firmicutes dominated the Outokumpu 500-m depth mica schist surfaces when incubated *in situ* (Nuppunen-Puputti et al., 2021). A major difference observed was the abundance of the *Pseudomonas* dominating in the microcosm conditions in contrast to lower relative abundance in the *in situ* incubated mica schist surfaces. Lack of hydrostatic pressure, its effect for microbial growth rates, and dissolution of gasses vs. closed system without constant gas exchange and potentially also accumulation of growth-limiting compounds are examples of the major differences microbial communities experience in microcosm compared to the natural subsurface environment. At the depth of 500 m, the hydrostatic pressure would reach 5 MPa. The lack of hydrostatic pressure in the microcosms could have enhanced the *Pseudomonas* growth rate and thus affect the community structure. Lack of gas exchange in the microcosms could also have slowed the growth rates for microbial groups dependent on dissolved gasses in their metabolism. However, in a study by Kaneko et al. (2000), a barotolerant *Pseudomonas* strain isolated from deep sea showed no major difference in growth rate between atmospheric and 10 MPa hydrostatic pressure (Kaneko et al., 2000). This would suggest drivers other than pressure for the observed difference in the abundance. In the future, pressurized enrichment experiments could clarify this issue further as we are missing obligate piezophiles from enrichments at normal atmospheric pressure. *Pseudomonas* strains generally have a quite versatile metabolism, which was also predicted for the OKU15 *Pseudomonas*, which had the potential to reduce nitrate, nitrite, and nitric oxide, thus contributing to the nitrogen cycling in the microbial community. In addition, OKU15 had genes for oxidation of methanol or formate, thus the potential for producing aldehydes and CO_2_. Our study thus further verifies previous results from Outokumpu that nitrate-reducing bacteria and especially *Pseudomonas* genus at 500 m depth were activated by added methane and/or methanol in Outokumpu deep subsurface fluid samples (Rajala et al., 2015a; Rajala and Bomberg, 2017). *Pseudomonas* bacteria have also been shown to dominate the active bacterial community in methane-rich groundwater in Olkiluoto at 415–572-m depths (Bomberg et al., 2015). Previously, we identified members of the Firmicutes phylum, i.e., *Dethiosulfatibacter*, unclassified *Comamonadaceae*, various Firmicutes, SRB2 group, *Proteiniclasticum*, unclassified *Rhodobacteraceae*, and unclassified *Acholeplasmataceae*, forming biofilm on mica schist surfaces *in situ* over 6 months at the depth of 500 m (Nuppunen-Puputti et al., 2021) with Actinobacteria and *Erysipelothrix* as minor groups. In the present study, we obtained MAGs affiliating with *Dethiosulfatibacter* 38–11 (OKU11) and *Proteiniphilum* (OKU04), in addition to three different *Acholeplasmataceae* (OKU06, OKU07, and OKU19) from the sessile community on the mica schist, confirming that these bacteria are a stable part of the deep biosphere biofilm community in the Outokumpu crystalline bedrock environment.

We also compared the development of the fungal community on mica schist between microcosms and previous *in situ* incubations. The main mica schist surface fungal phyla from the microcosms were the Ascomycota, Basidiomycota, and Mortierellomycota, which were similar to the fungi detected in the *in situ* incubation from the 500-m depth in Outokumpu (Nuppunen-Puputti et al., 2021). Mortierellomycota was only detected from the active fraction of the microcosms after 8 months of incubation. In addition, there were differences at the genus level as the long microcosm MSC enrichment favored *Aspergillus, Penicillium*, and *Acremonium*, whereas in the shorter *in situ* incubation, mica schist surfaces were dominated with, e.g., *Mortierella, Debaryomyces, Vishniacozyma*, and *Cladosporium.* In contrast, *Aspergillus* was merely detected as a minor genus in the *in situ* incubation at 500 m depth (Nuppunen-Puputti et al., 2021). In the mica schist microcosms, *Vishniacozyma* and *Cladosporium* were not detected from all samples but present on a few mica schist surfaces (Supplementary Table 1). In accordance to previous samplings from deep crystalline bedrock biosphere, the fungal communities tend to vary across samples. Microcosm environment supported mainly filamentous fungi, whereas the subsurface *in situ* conditions enriched more sessile yeasts on mica schist surfaces. Microcosms with MSC also hosted more diverse fungal communities at both time points compared to MSS microcosms.

### Attachment Strategies of Sessile Bacteria on Mineral Surfaces

Microbial rock surface attachment strategies including production of various appendices like stalks, hyphae-like tubular structures, and slime-like potential extracellular polymeric substances (EPS) were visualized with SEM. In detail, the microbial communities produced long tubular structures expanding over tens to hundreds of micrometers and showed microbial “hair-like” appendices potentially enabling better attachment to rock surfaces (Figures 1E,H). For example, Actinobacteria, Chloroflexi, and some Firmicutes represent potential filamentous structures forming taxa detected in these rock surface amplicons (Nielsen et al., 2009; Cao et al., 2016). The microbial rock surface community contained Actinobacteria belonging to Coriobacteriia, confirmed by both amplicon sequencing and metagenomic analyses (OKU08 and OKU14). Actinobacteria have been shown to produce tubular hyphae-like filamentous cell structures and to form basaltic rock weathering biofilms in oxic conditions (Cockell et al., 2013; Cao et al., 2016).

The genetic potential for biofilm formation in MAGs showed similarities for *Pseudomonas*’ Wsp-system especially in seven MAGs and potential ability to produce polysaccharides such as starch and glycogen across even more MAGs. The Wsp-system regulates the initiation of biofilm through feedback on motility gene expression (Hickman et al., 2005; Guttenplan and Kearns, 2013). Multiple MAGs had motility-linked genes for production of flagella and chemotaxis that are essential for reaching surfaces yet need to be downregulated in biofilms (Guttenplan and Kearns, 2013). GGDEF responsible for production of motility inhibiting c-di-GMP thus linked to the initiation of biofilms was detected in three MAGs (OKU8, OKU10, and OKU13). This further confirms that these deep subsurface mica schist surface microbial communities host genetic potential for biofilm formation to inhabit these rocky surfaces. This extracellular hair-structure formation could be linked to cellular metabolism, as sulfate-reducing bacteria (SRB) have been shown to produce hair-like nanowire structures under carbon-poor growth conditions, enabling the utilization of metal surfaces and potential use of extracellular electron transfer (EET) (Sherar et al., 2011). This would allow microorganisms to use mineral surfaces as electron source or sink (Vaughan and Lloyd, 2011; Ng et al., 2016; Deng et al., 2018, 2020). In addition, some microbial cells were covered with pellets, which could be membrane vesicle-like structures (Figure 1B). Membrane vesicles are common for many bacteria and have widely diverse functions, such as specialized “organ-like” parts, which may have, e.g., proton-pump activity, and can be used for distribution of hydrophobic signaling molecules, mineralization of toxins, accumulation of various elements when colonizing rock surfaces, and cell surface shedding linked to the reduction of metals and weathering, e.g., black shale (Matlakowska and Sklodowska, 2011; Manning and Kuehn, 2013; Shao et al., 2014; Toyofuku et al., 2015; Keren et al., 2017; Brameyer et al., 2018; Kwon et al., 2019).

## Conclusion

*Pseudomonas* was the cosmopolitan bacterial genus enriched in mica schist microcosms along with *Desulfosporosinus, Hydrogenophaga*, and *Brevundimonas.* Mica schist supported epilithic sulfate reducers and microbial cells showing various ways to enable attachment and interaction with mineral surfaces. Here, the observed attachment strategies included stalk formation, production of slime-like extracellular matrix, long tubular cell structures, and hair-like cell appendices as well as genes linked to biofilm formation. Mica schist microbial community metagenomes displayed a vast genetic metabolic potential ranging from wide metabolic capabilities to specialist lifestyles. Heterotrophy appears to be more common than autotrophy in the epilithic community MAGs. MAGs affiliating with major mica schist microbial community groups would potentially specialize in sulfur cycling, whereas minor group representing MAGs had genetic potential not only for a fermentative lifestyle, but also for carbon monoxide, arsenate, and selenate cycling. Acholeplasma MAGs contained potential tools for saprotrophic dead biomass scavenging lifestyle in deep continental biosphere. Attached microbial communities formed in deep subsurface microcosms showed both similarities and major differences in comparison to the previous sessile 500-m depth *in situ* mica schist communities. Mica schist supported sulfate-reducing bacteria, Firmicutes, Actinobacteria, and Acholeplasma in both enrichment experiments, whereas microcosm dominating *Pseudomonas* was only a minor group in the previous *in situ* experiment. Our results are in line with the previously suggested strong interaction between the deep subsurface microbial communities and the rock surfaces and that this interaction is crucial for sustaining life in the challenging anoxic and oligotrophic deep subsurface of crystalline bedrock environment. Furthermore, we encourage future research to combine studies of both planktic and sessile forms of microbial life to achieve a comprehensive view of the metabolic functions and interaction of the deep biosphere.

## Data Availability Statement

The sequence datasets presented in this study can be found under project accession number PRJEB48900 in the European Nucleotide Archive (ENA) at https://www.ebi.ac.uk/ena/.

## Author Contributions

MN-P and MB designed the experiments, did the data visualization, and prepared the original manuscript. MN-P performed the laboratory analyses with contribution from LP. MR and AS performed the SEM analysis. MN-P did the formal analysis. RK contributed with the geochemical data. IK provided funding and field site management. MB was the PI. The manuscript was edited and discussed with all authors.

## Conflict of Interest

MN-P, MB, and AS were employed by VTT Technical Research Centre of Finland Ltd. The remaining authors declare that the research was conducted with the highest academic integrity and without commercial interests.

## Publisher’s Note

All claims expressed in this article are solely those of the authors and do not necessarily represent those of their affiliated organizations, or those of the publisher, the editors and the reviewers. Any product that may be evaluated in this article, or claim that may be made by its manufacturer, is not guaranteed or endorsed by the publisher.

## Footnotes

1
https://www.icdp-online.org/projects/world/europe/outokumpu-finland

## Supplementary Material

Supplementary Table 2 Bacterial **(A)** and fungal community **(B)** ASV counts across samples.

Supplementary Table 7 MagicLamp hits for LithoGenie **(A)**, FeGenie **(B)**, WspGenie **(C)** and MnGenie **(D)**.

Supplementary Table 8 Anvi’o Kegg Kofams hits **(A)** and modules **(B)** for analysis in the Keggdecoder.

Supplementary Table 9 METABOLIC analysis results.
