# Supplementary material for: Epilithic Microbial Community Functionality in Deep Oligotrophic Continental Bedrock
Source: Front Microbiol. 2022 Mar 1;13:826048. doi: 10.3389/fmicb.2022.826048 (PMC8921683; doi:10.3389/fmicb.2022.826048)
Supplement: Supplementary file 5 [file Data_Sheet_1.PDF]

Supplementary Material

Supplementary material includes this document with additional information on geochemistry, additional figures for the amplicon sequence taxonomic affiliations, additional MnGenie analysis for the MAGs, sample sums for the amplicon sequenced bacterial and fungal communities, additional PCoA plots for the amplicon sequenced microbial communities, and alpha diversity estimates for the bacterial and fungal communities. In addition, supplementary material includes four separate data sheets/tables for the ASV counts, MagicLamp, Anvi'o called Kegg Kofams and module, and METABOLIC analyses.

Supplementary Table 1. Geochemistry of the mica schist and deep groundwater from the Outokumpu Deep Drill Hole used in the microcosms. Composition of the mica schist is from the database of Västi (2011) and groundwater composition is the average of two samples (OKU500-8 and OKU500-9) reported in Kietäväinen (2017).

Mica schist			Groundwater		
Depth	506.1	m	Depth	500	m
Ag	0.03	mg/kg	Ag	<0.01	µg/L
As	0.9	mg/kg	As	1.52	µg/L
Cd	0.05	mg/kg	Cd	0.10	µg/L
Pb	5.3	mg/kg	Pb	3.63	µg/L
Au	<2	µg/kg	B	120	µg/L
Bi	141.0	µg/kg	Bi	<0.02	µg/L
Sb	14.2	µg/kg	Sb	0.21	µg/L
Se	93	µg/kg	Se	<10	µg/L
Te	10.9	µg/kg	Be	<1	µg/L
LOI*	2.0	%	pH	6.87	
Na_2O	3.19	%	Na	1810	mg/L
MgO	2.38	%	Mg	12.8	mg/L
Al_2O_3	13.5	%	Al	<100	µg/L
SiO_2	68.3	%	Si	1.55	mg/L
P_2O_5	0.163	%	P	<50	µg/L
K_2O	2.27	%	K	18.6	mg/L
CaO	2.34	%	Ca	2245	mg/L
TiO_2	0.724	%	Li	93.5	µg/L
MnO	0.059	%	Mn	25.8	µg/L
Fe_2O_3	5.86	%	Fe_{tot}	<0.03	mg/L
S	0.057	%	S_{tot}	3.36	mg/L
Cl	0.006	%	Cl	7955	mg/L
Cr	0.0109	%	Cr	<2	µg/L
Ni	0.0047	%	Ni	2.82	µg/L
Cu	<0.002	%	Cu	<1	µg/L
Zn	0.0079	%	Zn	10.43	µg/L
Ga	0.0024	%	Tl	<0.02	µg/L
Sr	0.0186	%	Sr	22.05	mg/L
Mo	<0.001	%	Mo	17.3	µg/L
Sn	<0.002	%	Br	<50	mg/L
Ba	0.0479	%	Ba	1.61	mg/L
Co	14.1	mg/kg	Co	0.30	µg/L
Hf	4.49	mg/kg	I	1095	µg/L
Nb	8.45	mg/kg	F	<50	mg/L
Rb	79.1	mg/kg	Rb	38.6	µg/L
Ta	0.63	mg/kg	SO_4	<50	mg/L
Th	9.28	mg/kg	Th	<0.04	µg/L
U	2.47	mg/kg	U	<0.02	µg/L
V	102	mg/kg	V	0.65	µg/L
Zr	166	mg/kg	NO_3	<100	mg/L
C	0.42	%	Alkalinity	0.18	mmol/L

* Loss on ignition

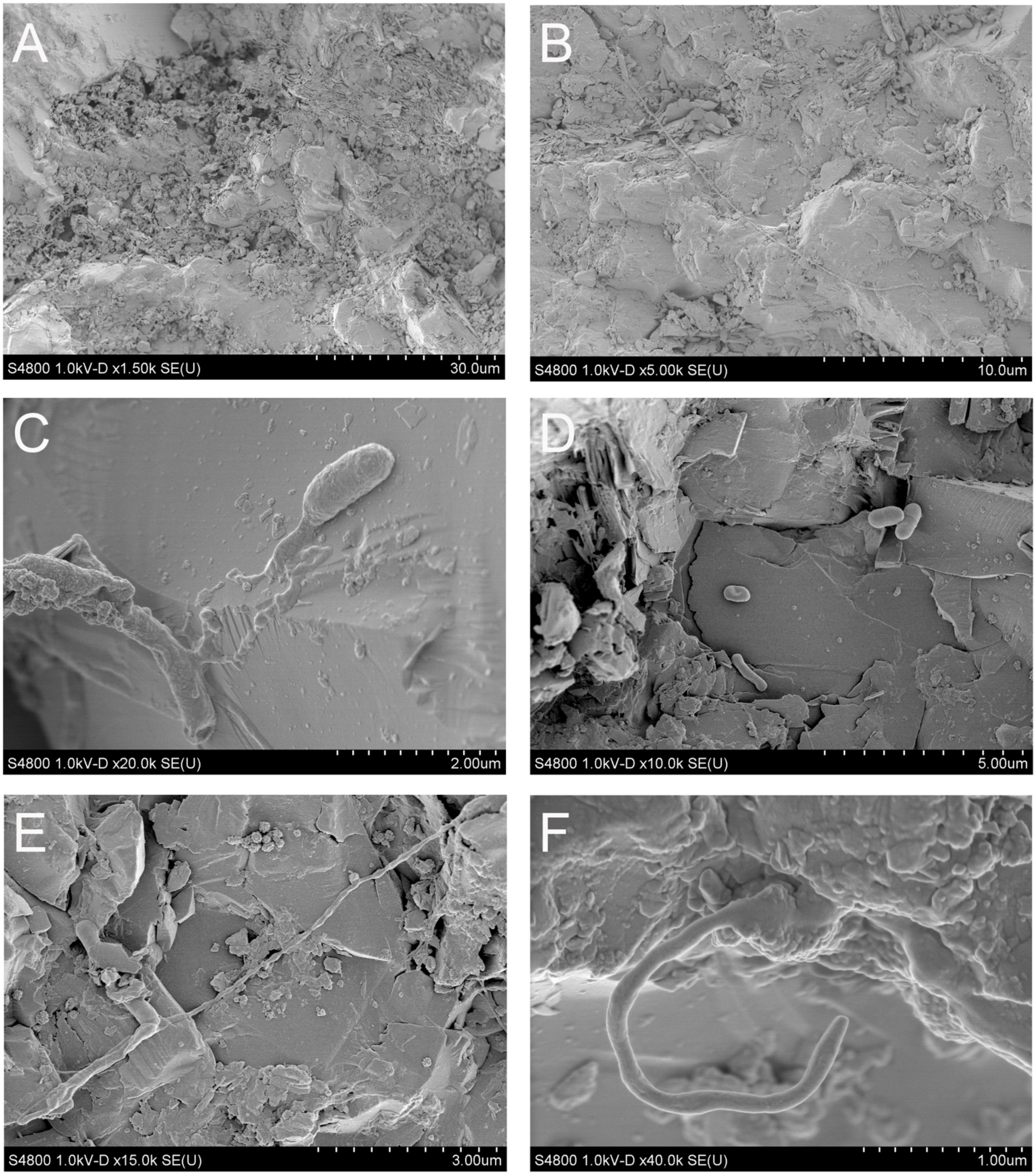

Supplementary Figure 1 A-F. Scanning electron microscopy images from the mica schist slides after eight months incubation. S1A: Long tubular cell-like structures expanding over tens of micrometers on mica schist surfaces, S1B: putative bacteria with long stalk, S1C: rod cell-like structure with potential EPS on mica schist surface, S1D: various different cell-types, S1E-F: tubular putative microbial cells.

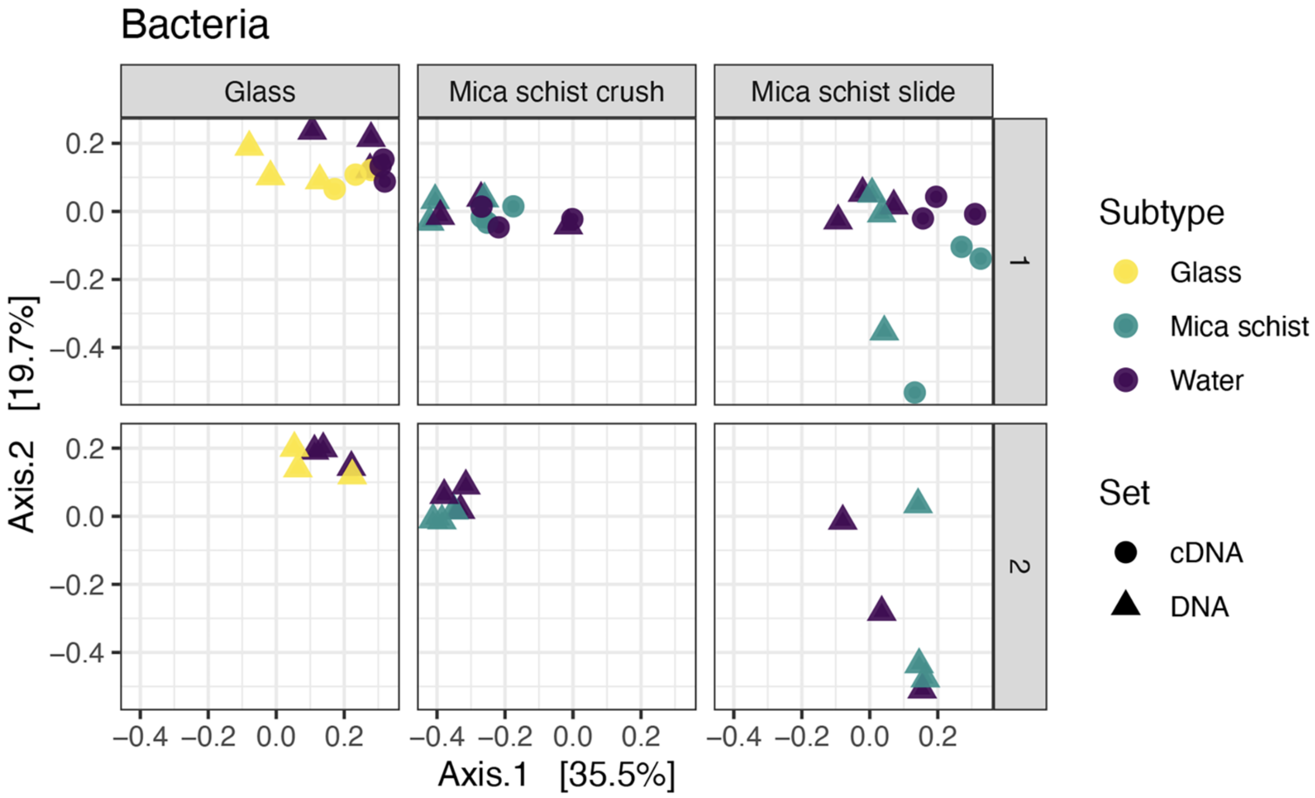

Supplementary Figure 2. Principal coordinates analysis for the active (cDNA) and total (DNA) bacterial communities after 8 and for the total (DNA) after 40 months of incubation in different sample types. Each subtype is described with different colors as shown in the legend to the right. Axis 1 describes 35.5% of the variance while Axis 2 describes 19.7% of the variance.

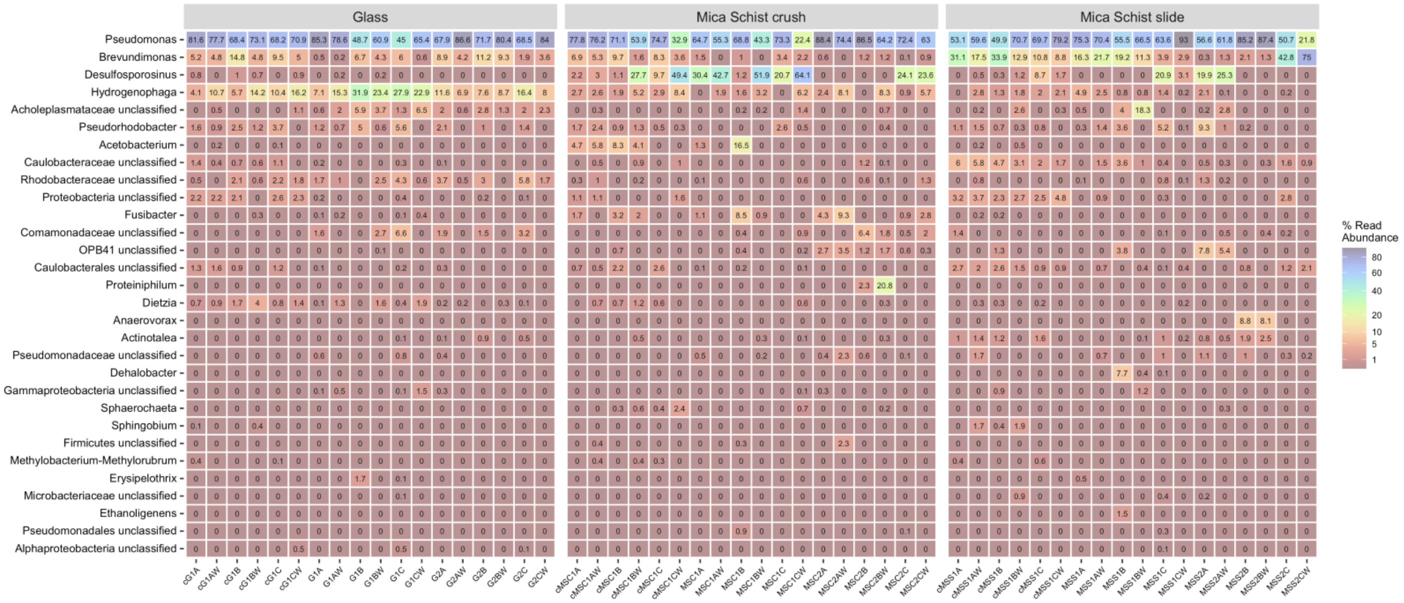

Supplementary Figure 3. Abundances of top 30 bacterial genera as percentages of reads across different sample types.

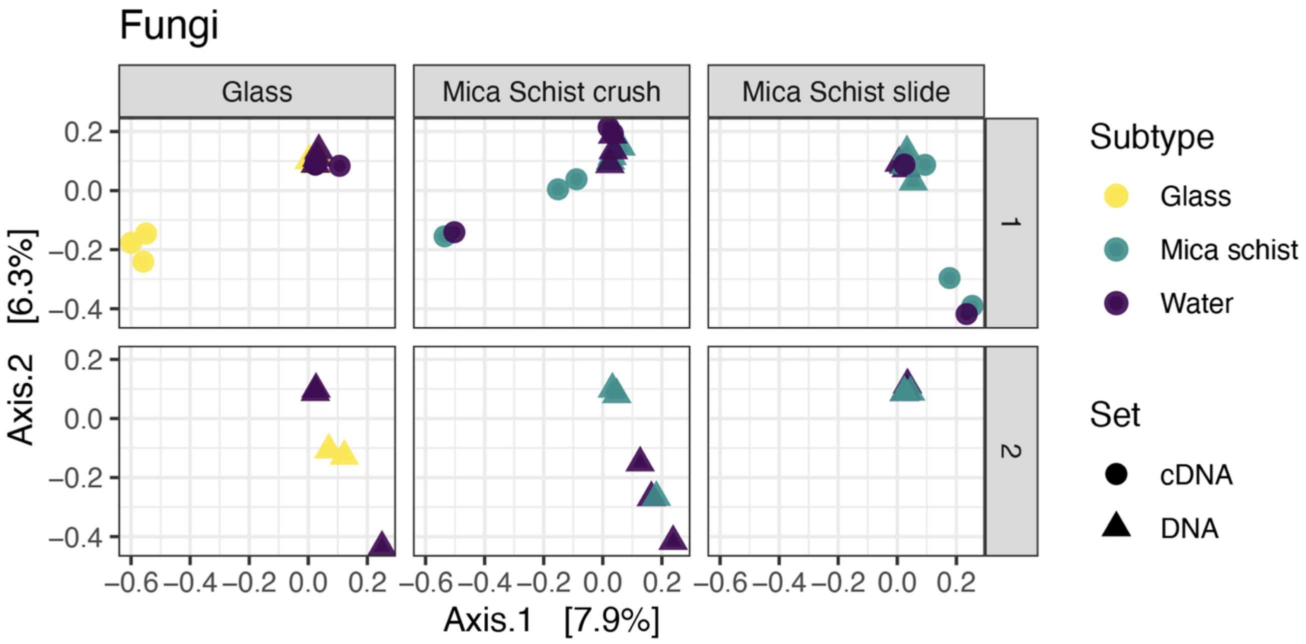

Supplementary Figure 4. Principal coordinates analysis for the fungal communities in DNA (triangle) and cDNA (dot) samples at two different time points 1 (8 months) and 2 (40 months) and in different sample types. Each subtype is described with different colors according to the legend on the right. Axis 1 describes 7.9 % of the variance while Axis 2 describes 6.3% of the variance.

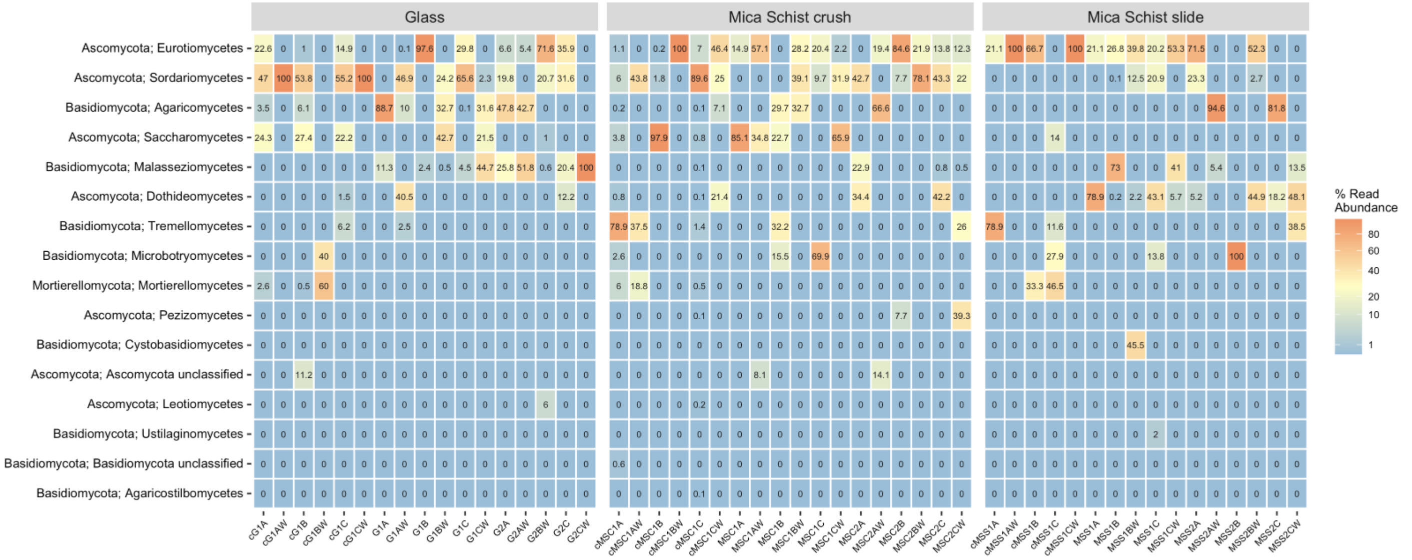

Supplementary Figure 5. Relative abundances (%) of all detected 16 fungal classes across different samples.

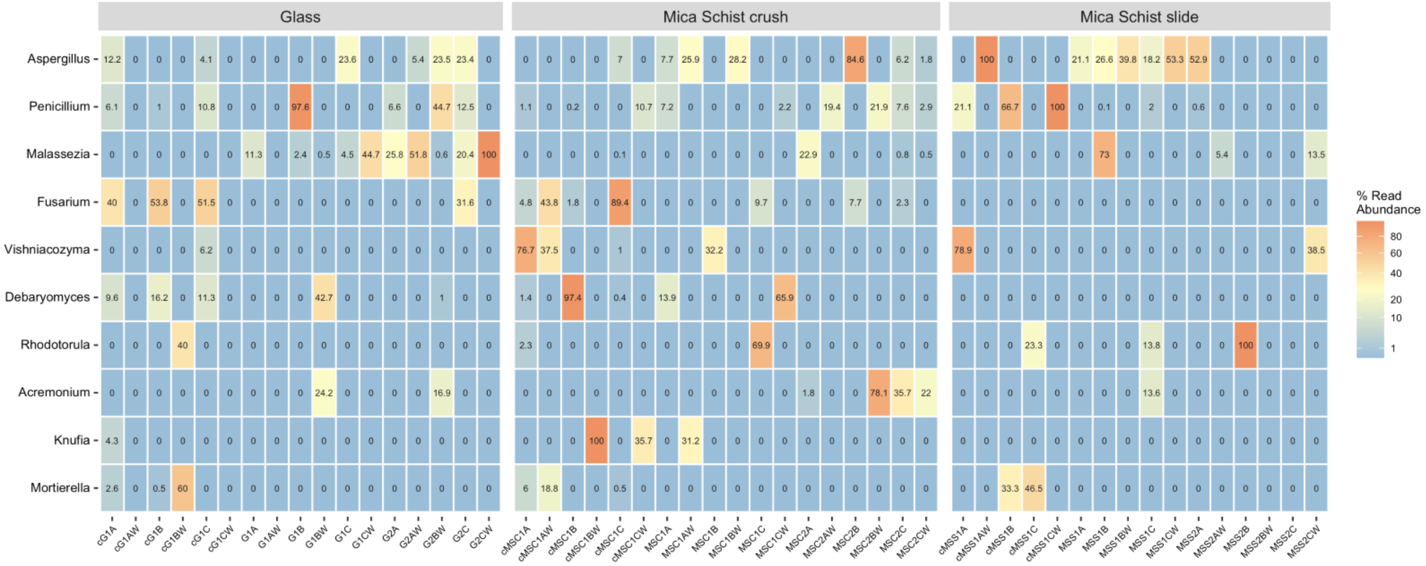

Supplementary Figure 6. Top 10 most abundant fungal genera across samples.

Supplementary Table 3. Sample sums for bacterial and fungal amplicon sequence samples in DNA and cDNA samples in both time points across sample types.

DNA, Time 1			cDNA, Time 1			DNA, Time 2		
**Sample ID**	**Bacteria**	**Fungi**	**Sample ID**	**Bacteria**	**Fungi**	**Sample ID**	**Bacteria**	**Fungi**
MSS1neg	13	398	cMSS1neg	896	1	MSS2neg	888	301
MSS1A	3501	71	cMSS1A	2265	19	MSS2A	1771	172
MSS1B	728	2111	cMSS1B	1890	6	MSS2B	486	73
MSS1C	8707	450	cMSS1C	1585	43	MSS2C	1902	77
MSS1AW	1340	736	cMSS1AW	1681	2	MSS2AW	2043	56
MSS1BW	831	279	cMSS1BW	1377	279	MSS2BW	965	661
MSS1CW	1662	953	cMSS1CW	1264	2	MSS2BCW	569	52
MSC1neg	10	6616	cMSC1neg	463	2113	MSC2neg	7	80
MSC1A	964	958	cMSC1A	922	1989	MSC2A	3154	218
MSC1B	3852	2242	cMSC1B	743	3003	MSC2B	171	13
MSC1C	116	103	cMSC1C	794	9405	MSC2C	3011	1314
MSC1AW	103	19965	cMSC1AW	1311	16	MSC2AW	86	434
MSC1BW	651	982	cMSC1BW	988	9	MSC2BW	1856	192
MSC1CW	2666	91	cMSC1CW	617	28	MSC2CW	690	17801
G1neg	9	610	cG1neg	1079	610	G2neg	606	0
G1A	78230	2815	cG1A	1039	115	G2A	2052	724
G1B	119	123	cG1B	1499	197	G2B	1206	0
G1C	11401	3638	cG1C	1540	194	G2C	3718	14571
G1AW	1277	121447	cG1AW	992	6	G2AW	993	2228
G1BW	962	3301	cG1BW	1115	5	G2BW	378	11955
G1CW	463	85886	cG1CW	797	4	G2CW	749	250

Supplementary Table 4. Observed number of ASVs, Chao1 richness estimate and Shannon diversity indices for the bacterial equimolarly pooled iSeq100 amplicon libraries.

Bacteria

	Observed	Chao1	se.chao1	Shannon
G1C	46	46	0.08	2.3
MSC1neg	5	6	1.26	1.5
MSC1A	15	16	1.81	1.2
MSC1B	17	17	0.49	1.2
MSS1AW	15	15	0.24	1.4
MSS1BW	13	13	0.48	1.6
MSS1CW	18	21	4.56	1.7
G1CW	12	12	0.00	1.7
MSC1AW	3	3	0.00	0.8
MSC1BW	11	12	1.28	1.7
MSC1CW	21	21	0.00	1.9
G2AW	11	11	0.00	1.4
G2BW	8	8	0.47	1.4
G2CW	11	11	0.00	1.6
MSC2neg	3	3	0.41	1.0
MSC2BW	16	16	0.24	1.3
MSC2CW	16	16	0.24	1.5
G2A	22	22	0.00	1.9
G2B	15	15	0.00	1.9
G2C	20	20	0.24	1.9
MSC2A	19	19	0.16	0.9
MSC2B	9	9	0.73	0.8
MSS1neg	2	2	0.00	0.7
MSC2C	21	21	0.12	1.1
MSS2AW	23	23	0.24	2.1
MSS2CW	8	8	0.47	1.0
MSS2A	22	22	0.00	2.1
MSS2C	21	21	0.00	1.9
MSS1C	43	43	0.12	2.5
G1A	75	75	0.50	1.6
G1B	8	8	0.00	1.7
MSC1C	7	7	0.00	1.3
G1AW	16	16	0.00	1.6
G1BW	19	19	0.24	2.4
G2neg	18	18	0.49	2.3
MSC2AW	7	7	0.00	1.2
MSS2neg	5	5	0.00	1.0
MSS2BW	14	14	0.00	2.1
MSS2B	14	14	0.48	2.1
MSS1A	17	17	0.00	1.6
MSS1B	12	12	0.00	1.9
G1neg	2	2	0.00	0.7
cMSS1neg	24	24	0.00	2.6
cMSS1A	16	16	0.00	2.0
cMSS1B	23	23	0.00	2.0
cMSS1C	23	23	0.00	2.3
cG1neg	26	26	0.00	2.5
cG1A	18	18	0.49	1.7
cG1B	20	20	0.49	2.2
cG1C	20	20	0.16	2.1
cMSC1neg	18	18	0.00	2.5
cMSC1A	19	19	0.00	1.5
cMSC1B	13	13	0.00	1.4
cMSC1C	17	17	0.00	1.6
cMSS1AW	28	28	0.00	2.3
cMSS1BW	24	24	0.00	2.1
cMSS1CW	20	20	0.00	2.0
cG1AW	16	16	0.00	1.7
cG1BW	18	18	0.00	2.2
cG1CW	20	20	0.00	1.9
cMSC1AW	22	22	0.00	1.7
cMSC1BW	17	17	0.00	1.8
cMSC1CW	15	15	0.00	2.0

Supplementary Table 5. Observed number of ASVs, Chao1 richness estimate and Shannon diversity indices for the fungal equimolarly pooled iSeq100 amplicon libraries.

Fungi

	Observed	Chao1	se.chao1	Shannon
G1A	3	3	0.0	0.9
G1B	3	3	0.0	0.8
G1C	5	5	0.0	0.9
MSC1neg	14	14	0.0	1.7
MSC1A	6	6	0.0	1.4
MSC1B	5	5	0.0	1.6
MSC1C	3	3	0.0	0.8
MSS1AW	10	10	0.2	1.2
MSS1BW	5	5	0.4	1.1
MSS1CW	4	4	0.0	0.9
G1AW	25	25	0.7	1.8
G1BW	6	6	0.0	1.4
G1CW	19	19	0.0	1.7
MSC1AW	7	7	0.0	1.6
MSC1BW	5	5	0.0	1.5
MSC1CW	3	3	0.0	0.7
G2neg	2	2	0.0	0.7
G2AW	4	4	0.0	0.9
G2BW	24	24	0.0	2.3
G2CW	1	1	0.0	0.0
MSC2neg	5	5	0.0	1.3
MSC2AW	5	5	0.0	1.3
MSC2BW	2	2	0.0	0.5
MSC2CW	14	15	2.3	1.7
G2A	6	6	0.0	1.4
G2C	19	19	0.9	2.3
MSC2A	4	4	0.0	1.1
MSC2B	4	5	2.2	1.1
MSS1neg	12	12	0.0	2.3
MSC2C	12	12	0.0	1.7
MSS2neg	2	2	0.0	0.3
MSS2AW	2	2	0.0	0.2
MSS2BW	4	4	0.0	1.1
MSS2CW	3	3	0.0	1.0
MSS2A	8	8	0.5	1.6
MSS2B	1	1	0.0	0.0
MSS2C	3	3	0.4	0.5
MSS1A	2	2	0.0	0.5
MSS1B	6	6	0.0	0.8
MSS1C	12	12	0.0	2.2
G1neg	8	8	0.0	1.3
cMSS1neg	1	1	0.0	0.0
cMSS1A	2	2	0.0	0.5
cMSS1B	2	2	0.0	0.6
cMSS1C	6	6	0.0	1.7
cG1neg	6	6	0.0	1.6
cG1A	11	11	0.2	1.9
cG1B	7	7	0.2	1.4
cG1C	9	9	0.0	1.6
cMSC1neg	8	8	0.0	0.3
cMSC1A	25	25	0.0	2.0
cMSC1B	5	5	0.4	0.1
cMSC1C	26	26	0.0	1.3
cMSS1AW	1	1	0.0	0.0
cMSS1CW	1	1	0.0	0.0
cG1AW	1	1	0.0	0.0
cG1BW	3	3	0.1	1.1
cG1CW	1	1	0.0	0.0
cMSC1AW	3	3	0.0	1.0
cMSC1BW	1	1	0.0	0.0
cMSC1CW	5	5	0.0	1.5

Supplementary Table 6. Assembly statistics for the MEGAHIT co-assembly from the MetaQuast analysis. Num is an abbreviation for "Number of", kbp for kilo base pairs and bp for base pairs.

Assembly statistic	MEGAHIT co-assembly
Genome fraction (%)	54.009
Duplication ratio	1.16
Largest alignment	126957
Largest contig	126957
Largest contig without reference	285919
Missassembled contig length	8103126
Num contigs without reference	36446
Num indels per 100kbp	81.63
Num misassemblies	3105
Num Ns per 100kbp	0
Num of mismatches per 100 kbp	2853.77
Total aligned length	21442209
Total lenght	157850493
Total length (1000 bp)	157850493
Total length (10 000 bp)	73291914
Total length (50 000 bp)	20553281

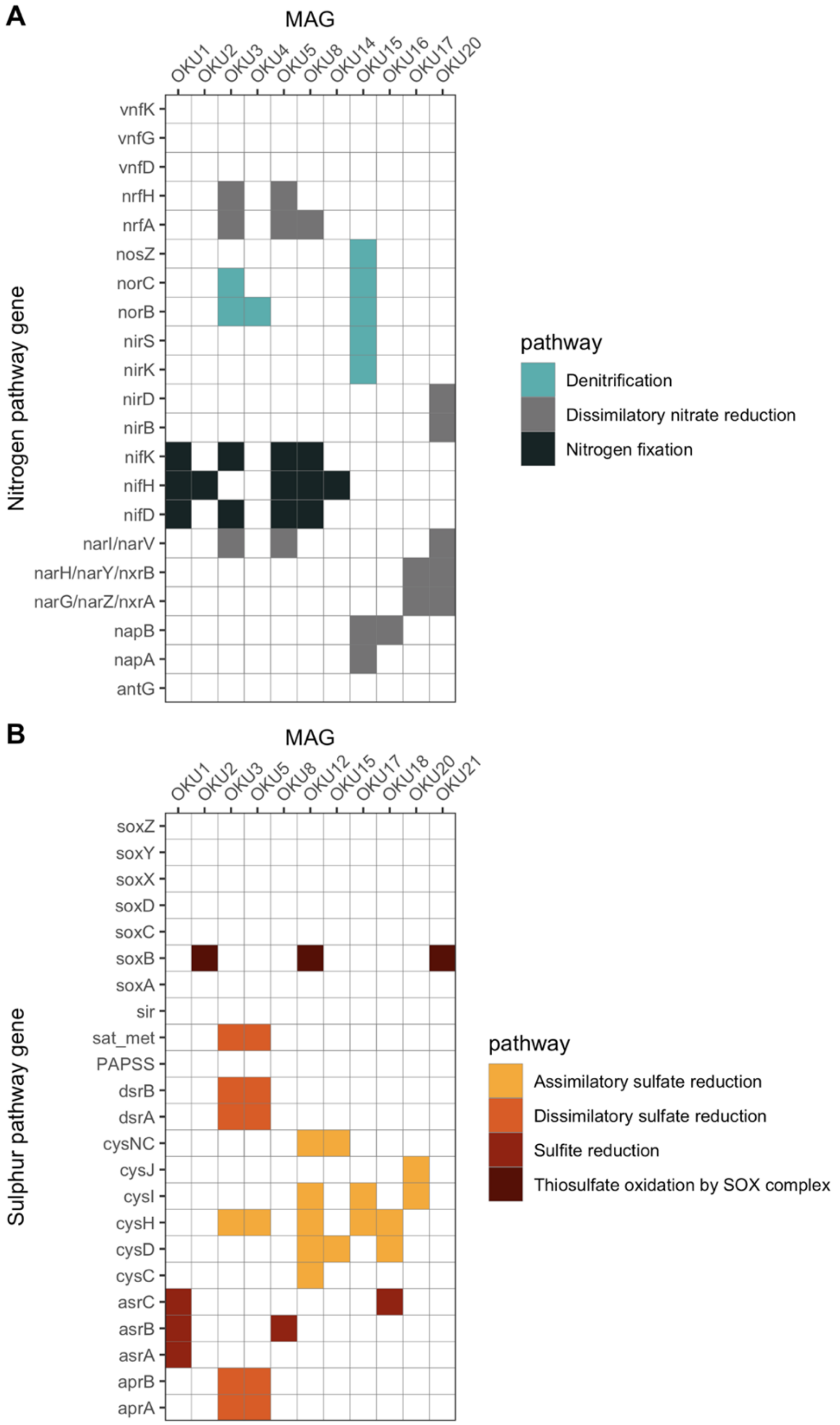

Supplementary Figure 7. Nitrogen and sulphur pathways' genes in the presence-absence map. X-axis represents MAGs with Kegg kofams hits, and Y-axis represents gene name, color represents in which pathway genes belongs to.

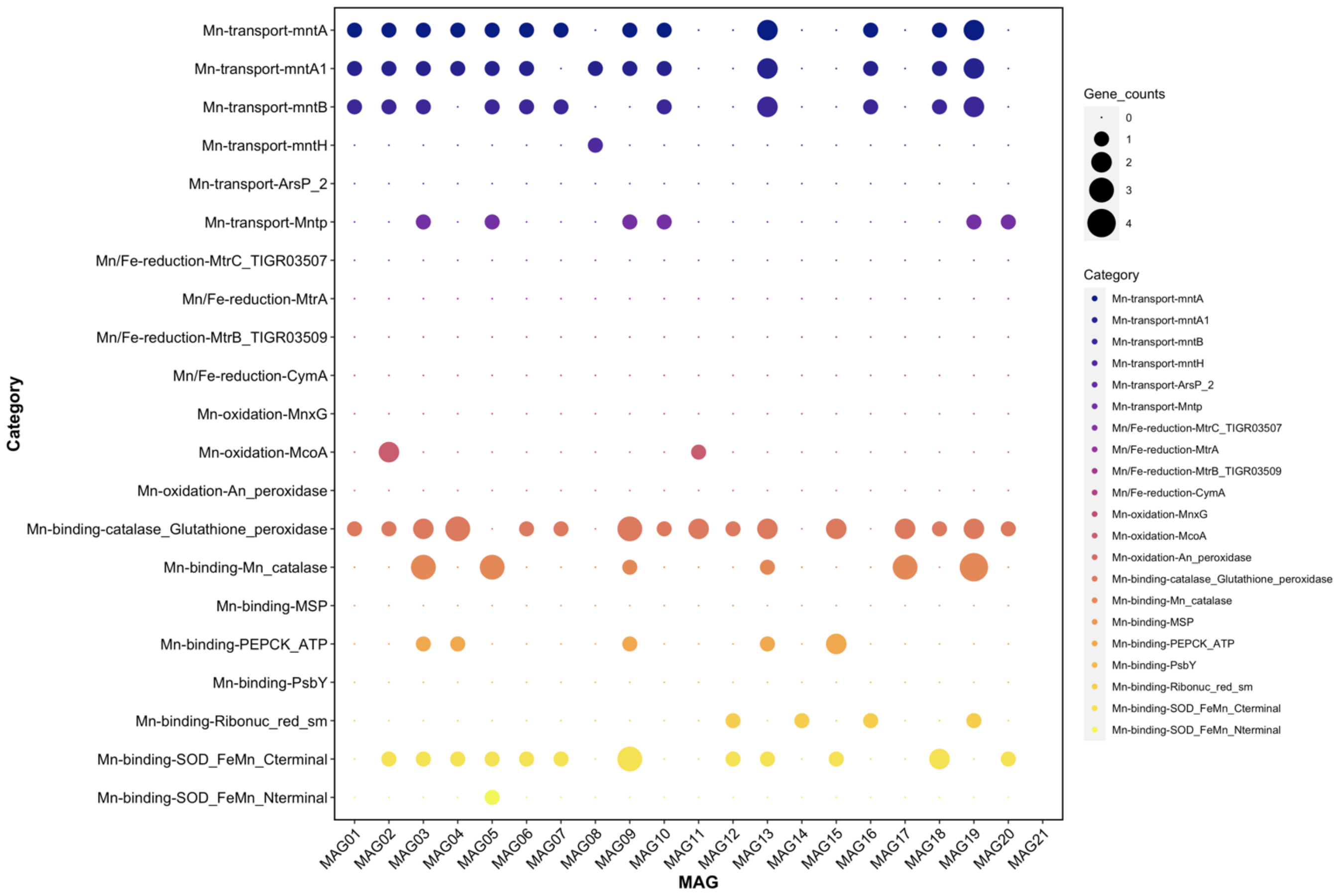

Supplementary Figure 8. MnGenie heatmap for Mn transport, Mn oxidation and Mn-binding enzymes linked hmm genes normalized to total gene count in Sample contigs.
